# Major Sensory Attributes and Volatile Compounds of Korean Rice Liquor (*yakju*) Affecting Overall Acceptance by Young Consumers

**DOI:** 10.3390/foods9060722

**Published:** 2020-06-02

**Authors:** JeongAe Heo, Han Sub Kwak, Miran Kim, Jae-Ho Kim, Hyung Hee Baek, Hyukjin Shin, Young-seung Lee, Sanghyeok Lee, Sang Sook Kim

**Affiliations:** 1 Technical Assistance Center, Korea Food Research Institute, Wanju-gun 55365, Korea; heo.jeongae@kfri.re.kr; 2 Research Group of Food Processing, Korea Food Research Institute, Wanju-gun 55365, Korea; hskwak@kfri.re.kr (H.S.K.); ranni1027@kfri.re.kr (M.K.); 3 Planning Division, Korea Food Research Institute, Wanju-gun 55365, Korea; ricewine@kfri.re.kr; 4 Department of Food Engineering, Dankook University, Cheonan 31116, Korea; baek@dankook.ac.kr (H.H.B.); scoyy2001@naver.com (H.S.); 5 Department of Food Science and Nutrition, Dankook University, Cheonan 31116, Korea; youngslee@dankook.ac.kr (Y.-s.L.); leesh940324@gmail.com (S.L.)

**Keywords:** descriptive analysis, Korean rice liquor, SAFE-GC/MS, *yakju*

## Abstract

The sensory characteristics and volatile compounds that affect consumers’ acceptance of rice liquors were investigated. A total of 80 consumers evaluated 12 *yakju* samples and descriptive analysis by 11 trained panelists was conducted. Solvent-assisted flavor evaporation-gas chromatography-mass spectrometry analysis also was conducted revealing 120 volatile compounds in the *yakju* samples. Sensory attributes (*n* = 31) except appearance attributes were used for principal component analysis (PCA). As results, fruit odor (apple, hawthorn, omija, and pineapple odor) and flower odor (chrysanthemum, pine, and peppermint odor) were placed on the positive side of PC1 whereas persimmon vinegar odor, bitter taste, alcohol flavor, stinging and coating mouthfeel were located on the negative side of PC1. The *yakju* samples were mainly characterized by their alcohol content and supplementary ingredients. Sensory descriptors (*n* = 31; except appearance attributes and *p* > 0.05) and volatile compounds (*n* = 30; *p* > 0.5 correlation coefficient with overall acceptance) were chosen for multiple factor analysis (MFA). The MFA correlation map showed that ethyl propanoate, ethyl-2-hydroxy-2-methylbutanoate, methyl 2-furoate, γ-butyrolactone, 4-ethoxycarbonyl-γ-butyrolactone, hawthorn odor, apple flavor, grape flavor, and sweet taste were positively correlated with young consumers’ overall acceptance. Additionally, negative correlation with overall acceptance was found in 1,3-butanediol, 2,3-butanediol, and 1,1-diethoxy-3-methylbutane.

## 1. Introduction

*Yakju* is a representative traditional Korean rice wine along with *makgeolli*. *Yakju* is made with rice as a starch source, water, and *nuruk*. *Nuruk* is a starter made from grains and it plays an important role in the flavor of *yakju* during fermentation because it contains various microorganisms, including fungi and wild types of yeast [1,2]. Thus, during fermentation, sugars, various organic acids, and numerous volatile compounds are produced, affecting the flavors of the *yakju* [3]. The sensory properties of *yakju* are affected by various factors, and they are influenced not only by the yeast strain [2] but also by the fermentation process and the starch ingredients [4], and by the degree of milling of the rice used for the *nuruk* [5]. In addition, supplementary ingredients, such as mulberry [6], *Codonopsis lanceolate* [7], *Ganoderma lucidum* [8], and buckwheat sprouts [9] are used to improve the flavor and biological activities of *yakju*.

Regarding the studies on *yakju*, Lee et al. [10] characterized five commercial rice wines containing supplementary ingredients through descriptive and physicochemical analyses. Additionally, Lee [11] reported the extrinsic factors, such as brand and familiarity, that affect the acceptance of *yakju*. However, limited information is available on the specific volatile compounds and sensory attributes associated with consumer acceptance of *yakju*.

Studies focusing on the characterization of liquors using instrumental analysis were recently conducted. For example, Kang et al. [12] discriminated traditional Korean liquor (*makgeolli* and *yakju*) and Japanese (sake samples) using solid-phase microextraction gas chromatography-mass spectrometry (SPME-GC/MS). Kang et al. [13] also discriminated Korean rice wine (*makgeolli*) samples using electronic tongues (e-tongues) and LC-MS/MS. However, the bitterness of the *makgeolli* samples caused by amino acids was not well predicted by the e-tongue in the study of Kang et al. [13]. Xiao et al. [14] reported on the aroma profiles of three types of liquor (strong/light/sauce odor type) using GC-MS. They characterized the samples based on sensory attributes and volatile compounds through partial least squares (PLS) regression. Xiao et al. [15] also conducted a sensory evaluation as well as an instrumental analysis of liquors, and they investigated the correlation between sensory attributes and volatile compounds in cherry wines. They reported that volatile compounds such as 1-propanol, 2-ethyl-1-hexanol, geraniol, ethyl hexanoate, ethyl octanoate, and butanoic acid were positively correlated with sweet aroma (>0.9) in cherry wine samples.

A limited number of studies have focused on volatile compounds, sensory attributes, and consumers’ acceptance of *yakju* products containing various supplementary ingredients, which might produce complex and diverse flavors. Furthermore, Kim et al. [16] reported that young consumers in Korea tended to prefer soju (diluted liquor) and beer over the traditional liquor because of price, flavor, and hangover. To overcome the low acceptance of traditional liquor among young consumers, the key sensory attributes and volatile compounds that affect the acceptance of *yakju* containing supplementary ingredients must be identified. Therefore, this study was conducted to identify the key sensory attributes and volatile compounds that affect the acceptance of *yakju* containing supplementary ingredients by investigating the relationship of sensory attributes and volatile compounds with young consumers’ acceptance of *yakju*.

## 2. Materials and Methods

### 2.1. Yakju Samples

The 12 *yakju* samples used in this study were selected based on their availability in online stores and based on the specialty of the liquor-producing regions in Korea. Information on the ingredients of the products obtained from the label of each product, is shown in Table 1. The samples were refrigerated at 4 °C until they were used. 

### 2.2. Descriptive Analysis

Descriptive analysis and consumer test were approved by the institutional review board of Dankook University (DKU 2018-10-002). Eleven panelists (females, aged 37–49 years) were selected from 30 preliminary panelists based on their ability to discriminate and describe tastes and flavors in a screening test. These panelists participated in 15 training sessions (2 h per session, twice a week). During the panel training, 48 sensory descriptors (appearance = 3, odor = 17, taste/flavor = 13, mouthfeel = 6, and aftertaste/after-mouthfeel = 9), definitions, reference materials, and intensity of reference materials for the 12 *yakju* products were developed (Table 2). The samples were evaluated in an individual sensory booth equipped with a computerized data collection system (Korea Food Research Institute, Wanju-gun, Korea) using a 15 cm line scale (0: none to 15: very strong). The panelists evaluated six samples for each session, and they were given 15 min to test one sample. After testing three samples, they were required to rest for 15 min to prevent sensory fatigue. The *yakju* samples (40 g) were monadically presented in a glass cup (55 mL, diameter of top of cup = 5 cm) coded with a three-digit randomized number and were presented in a randomized order to prevent bias. Each sample was covered with a watch glass (7 cm in diameter) to minimize changes in odor during the evaluation. Filtered water and crackers were provided as palate cleansers. A spit cup was also provided for panelists to use when they did not want to swallow the samples. Evaluation sessions were conducted in three replications.

### 2.3. Consumer Test

All of the consumers who participated in this study were users of *yakju,* and they were recruited based on them having no allergies to alcohol or *yakju* and based on their willingness to participate in the study. A total of 80 consumers (in their 20 s; male = 38, female = 42) participated in the evaluations. The samples were served following the Williams Latin Square design. The participants evaluated the overall acceptance using a 9-point hedonic scale. Six samples were provided for one evaluation session. Each sample (30 mL) was served in a paper cup (70 mL), and filtered water was provided as a palate cleanser. The consumers were asked not to consume any food for at least 1 h before the evaluation.

### 2.4. Identification of Volatile Compounds by GC-MS

Prior to the GC-MS analysis, the volatile compounds of the samples were extracted using the liquid-liquid continuous extraction (LLCE)/solvent-assisted flavor evaporation (SAFE) method. Each sample (1150 mL) and 3-heptanol (328 μg; internal standard) were placed in LLCE apparatus and extracted for 8 h at room temperature using 300 mL of redistilled dichloromethane as an extraction solvent. The LLCE extracts were frozen for 12 h at −20 °C to remove water and then concentrated to 120 mL using a gentle N_2_ gas stream. SAFE was applied to remove non-volatile compounds and impurities, such as pigments. The extract (120 mL) was distilled for 30 min at 40 °C under 8.0 × 10^−3^ Pa, and the SAFE extract was frozen for 12 h at −20 °C and then dried over anhydrous sodium sulfate (3 g). This extract was concentrated to 500 μL using a gentle N_2_ gas stream and then analyzed by GC-MS.

Analyses were performed using an Agilent 7890B GC/Agilent 5977A mass selective detector (Agilent, Santa Clara, CA, USA) with a DB-wax column (60 m length × 0.25 mm i.d. × 0.25 µm film thickness; J&W Scientific, Folsom, CA, USA). Helium was used as a carrier gas with a flow rate of 1.0 mL/min. The oven temperature was initially set at 40 °C for 5 min and then increased at a rate of 5 °C/min to 200 °C, which was held for 20 min. The samples (1 µL) were injected into GC-MS apparatus at split mode (50:1). The injector and detector temperatures were 200 °C and 250 °C, respectively. The ionization voltage was 70 eV, and the mass range was 33–350 *m*/*z*. Analyses were conducted in triplicate.

Volatile compounds were identified based on their retention indices (RI), Wiley Registry of Mass Spectral Data (9th ed.) and by using a NIST08 database (Agilent). The concentration of the volatile compounds was semi-quantified using Formula (1), wherein the correlation coefficient of the peak area ratio and the amount ratio was assumed to be 1.

(1) $$\mathrm{Concentration}\;\left(\mathrm{ppb}\right)=\frac{\mathrm{Peak}\;\mathrm{area}\;\mathrm{ratio}\times µg\;\mathrm{of}\;3-\mathrm{heptanol}\;}{L\;\mathrm{of}\;\mathrm{samples}}$$

### 2.5. Statistical Analysis

The descriptive analysis results and the consumers’ acceptance data were analyzed by analysis of variance (ANOVA) to determine the differences among the samples. Student–Newman–Keuls (SNK) multiple comparison was used when a significant difference was found among the samples (*p* < 0.05). Principal component analysis (PCA) was conducted to summarize the results of the sensory characteristics of the *yakju* samples. Pearson’s correlation analysis was performed to investigate the relationship between consumers’ acceptance and volatile compounds. Moreover, multiple factor analysis (MFA) was performed to investigate the relationship among sensory attributes, volatile compounds, and consumers’ overall acceptance of the 12 *yakju* samples. All statistical analyses were conducted using XLSTAT (Ver. 2017.1, Addinsoft, Paris, France).

## 3. Results and Discussion

### 3.1. Descriptive Analysis of Yakju Samples

The results of the ANOVA showed significant differences in 33 attributes of the 48 attributes (Table 3). Among the samples, Y10 and Y8 showed the highest scores for redness and yellowness, respectively. The redness of Y10 might be due to the red color of the raw materials, such as the *Schizandra chinensis* fruit (omija), *Cornus officinalis,* and the *Lycium chinense* fruit. Similarly, some of the samples were characterized by attributes induced by their raw materials. For example, Y5, which contained chrysanthemum, had a strong chrysanthemum, peppermint, and pine odor. Y1 was highest in ginseng and hawthorn odor and Y7 had the highest score for bitterness and bitter aftertaste. This result might be due to saponin, which has a bitter taste [17]. Other ingredients such as fructose, also seemed to affect sweet taste in the Y2 and Y10 samples. In terms of alcohol flavor, samples with high alcohol contents such as Y7 (16.0%) and Y8 (18.0%) tended to be the highest in alcohol flavor, whereas Y10 (12.0%) and Y12 (11.0%), which contained relatively low alcohol content, were the lowest in alcohol flavor. Especially, Y8, which had the highest alcohol content, was the highest in body, coating and residue mouthfeel.

Y4 showed the highest scores for persimmon vinegar odor, sourness, stinging mouthfeel, and stinging aftertaste. This result is possibly due to the formation of acetic acid during fermentation, considering that acetic acid is associated with vinegar scent and a pungent odor [18].

**Figure 1:**
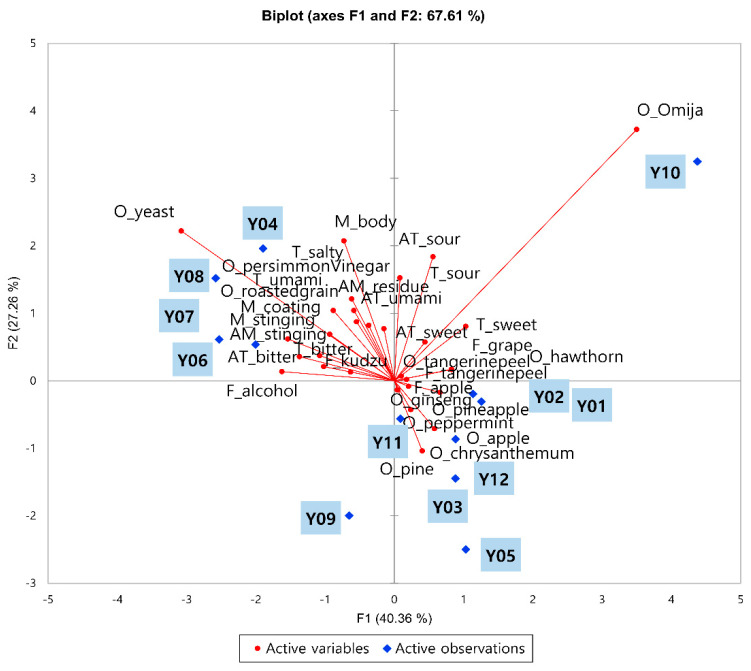
PCA plot of the 31 sensory descriptors of the 12 *yakju* samples. O, T, F, M, AT, and AM stand for odor, taste, flavor, mouthfeel, aftertaste, and after-mouthfeel, respectively.

The PCA result for the 31 sensory descriptive attributes of the 12 *yakju* samples is shown in Figure 1. Appearance-related descriptors (degree of clearness/redness/yellowness) were excluded to focus on the odor and flavor of the *yakju* samples, and attributes that had significant difference among the samples (*n* = 31) were used for the PCA. A total of 67.6% of variance could be explained by PC1 (40.4%) and PC2 (27.3%). Characteristics related to sweetness, flower, and fruit ingredients were located on the positive side of PC1, whereas bitter taste, burning mouthfeel, and alcohol odor and flavor were located on the negative side of PC1. Specifically, samples such as Y1, Y2, Y3, Y5, Y10, and Y12, which contained fruit or floral ingredients, were located on the positive side of PC1; these samples had *hawthorn* odor, sweet taste, sour taste, apple flavor, grape flavor, or tangerine peel odor/flavor. 

On the contrary, samples containing Supplementary Materials other than fruit or floral ingredients, such as sweet pumpkin (Y4), balloon flower root (Y6 and Y7), or soy bean (Y8), were located in quadrant 2. These samples were mainly characterized by yeast odor, roasted grain odor, alcohol flavor, and body and stinging attributes. Characteristics related to mouthfeel and taste were located on the positive side of PC2, whereas most of the attributes related to odor were located on the negative side of PC2. Particularly, salty taste, sour taste/aftertaste, and body mouthfeel were located on the positive side of PC2, whereas pine odor, peppermint odor, and chrysanthemum odor were located on the negative side of PC2. Samples with high alcohol levels were located on the positive side of PC2, whereas those with relatively low alcohol content, such as Y5 and Y12, were located on the negative side of PC2. Among the samples, Y9 was the only sample located in quadrant 3, and it had a low score for sour, sweet, umami, and fruit-related notes compared with other samples.

Overall, the present results indicated that the yakju samples were characterized mainly by their supplementary raw materials and alcohol content, which affect the overall odor and flavor of the samples.

### 3.2. Consumers’ Acceptance

The mean overall acceptance scores by 80 young consumers are shown in Table 4. Significant differences among the 12 samples were found in the overall acceptance scores. Overall acceptance was highest for Y3 (6.71), followed by Y10 (6.41), and Y2 (6.34) and was lowest for Y8 (3.25). Generally, overall acceptance was higher for samples with fruit-related supplementary ingredients (Y2, Y3, and Y10) than that for samples with root-related bitter ingredients (Y7 and Y8). Apparently, consumers preferred those samples with fruit-related characteristics over the bitter and yeasty samples. Similarly, Lee and Lee [19] studied the sensory attributes and acceptance of 10 *yakju* samples with supplementary ingredients, and they reported that the acceptance of various clusters of consumers were positively associated with fruit flavor, sweet aroma, and medicinal herb aroma but astringent mouthfeel, bitter taste, and yeast flavor were negatively associated with consumers’ acceptance. Moreover, Kwak et al. [20] reported that the key liking factors of rice wine by American panelists were sweet, sour, and apricot flavors, whereas the key disliking factors were yeasty and nutty characteristics. They reported positive correlations between overall acceptance and fruit-related characteristics (apple, peach, and pear), confirming the key liking factors for the *yakju* samples [20].

### 3.3. Volatile Compounds Identified Using GC/MS

A total of 120 volatile compounds (acids = 14, alcohols = 26, aldehydes = 2, esters = 32, furans = 2, ketones = 11, lactones = 7, phenols = 8, terpenoids = 6, and miscellaneous = 12) were identified (Table 5). Thirty-five volatile compounds were found in all of the samples.

Generally, acetic acid is responsible for the pungent odor in vinegar [18]. Huh et al. [21] reported that high acetic acid content could decrease the taste of a liquor, although the threshold for acetic acids is approximately 280,000 µg/L in a 10% ethanol system [22]. Among the 12 samples, Y4 had the highest acetic acid content (14,700 µg/L) followed by Y8 (5038 µg/L), while Y12 had the lowest acetic acid content (624 µg/L). Y7 was the only sample contained formic acid (11.3 µg/L) and 2-butenoic acid (12.4 µg/L). Fan and Qian [23] suggested that free fatty acids such as hexanoic acid and octanoic acid were produced by bacteria during the fermentation process. Y9 had the lowest content of 2-methyl propanoic acid (105 µg/L), butanoic acid (20.1 µg/L), 3-methyl butanoic acid (104 µg/L), and hexanoic acid (56.7 µg/L), which seemed to be associated with the degree of fermentation.

Alcohols, the largest group of volatile compounds in alcoholic beverages such as red wine [24,25], are produced during yeast metabolism, and alcohol content varies depending on the yeast starters [26]. Fusel alcohols, such as *n*-propyl alcohol, *iso*-butyl alcohol, and *iso*-amyl alcohol, are produced during the fermentation of amino acid in yeast [12,27]. Among them, isoamyl alcohol has the highest amount of the volatile compounds as reported by Kim et al. [28]. Isoamyl alcohol is known to boost aroma and flavor when it exists in low amounts [27]. Additionally, 2-methyl-1-propanol, which is one of the aliphatic alcohols related to alcohol odor [1], was high in Y1 (10,085 µg/L), Y2 (12,313 µg/L), and Y5 (10,222 µg/L).

Esters are produced during alcoholic fermentation by yeast; they have a fruit-like aroma and thus they positively influence the aroma quality of liquors [29,30,31]. The large number of volatile ester compounds (*n* = 32) could be explained by the various supplementary ingredients used to produce the samples. Isoamyl acetate is related to sweet and fruity aromas, and Mamede et al. [29] reported that low concentration of isoamyl acetate results in low consumers’ acceptance of sparkling wine samples. Isoamyl acetate content was highest in Y10 and lowest in Y8. Considering that overall acceptance of Y10 (6.41) was much higher than that of Y4 (3.25) as in Table 4, the results of this study confirmed that isoamyl acetate might be one of the key compounds affecting consumer acceptance. Among the samples, Y4 contained high amounts of ethyl lactate (45,468 μg/L), isoamyl lactate (446 μg/L), diethyl succinate (8015 μg/L), and ethyl hydrogen succinate (6056 μg/L). Apostolopoulou et al. [32] showed that the ethyl lactate content of samples of bottled Greek distillates (tsipouro) differs from that of homemade samples, suggesting that production methods might influence the amount of ethyl lactate. Argyri et al. [33] also analyzed the volatile compounds of meat samples under different temperatures. As the temperature increased (from 0 °C to 15 °C), the amount of ethyl lactate also increased. These results suggested that the ethyl lactate content of Y4 was affected by the manufacturing environment, such as production methods and temperature.

Ethyl octanoate contents in all of the samples was less than the threshold, which was reported to be 170 μg/L [34]. Y7 had the highest level of 2-phenylethyl acetate (759 μg/L), followed by Y6 (600 μg/L). This might be due to the specific yeast used in Y6 and Y7, considering that this compound is known to be formed during fermentation by yeast [34]. The intensity of 2-phenylethyl acetate in Y7 and Y6 was much higher than threshold (180 μg/L), which was reported in [34].

A high amount of terpenes is associated with a flower-like odor [35]. Given that eucalyptol, α-terpineol, and 4-terpinenol are found in Chrysanthemum morifolium R. [36], the five terpenes (eucalyptol, 4-terpineol, α-terpineol, borneol, and p-cymen-8-ol) found in Y5 might have originated from Chrysanthemum morifolium R., which was that sample’s major supplementary ingredient.

### 3.4. Relationship among Sensory Attributes, Volatile Compounds and Consumers’ Acceptance of Yakju Samples by MFA

**Figure 2:**
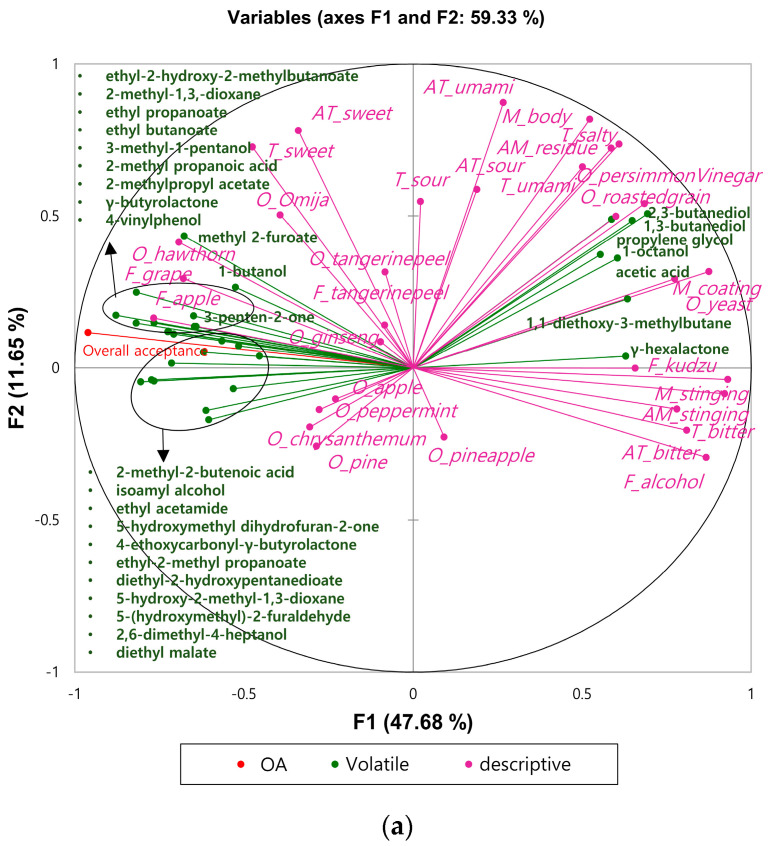

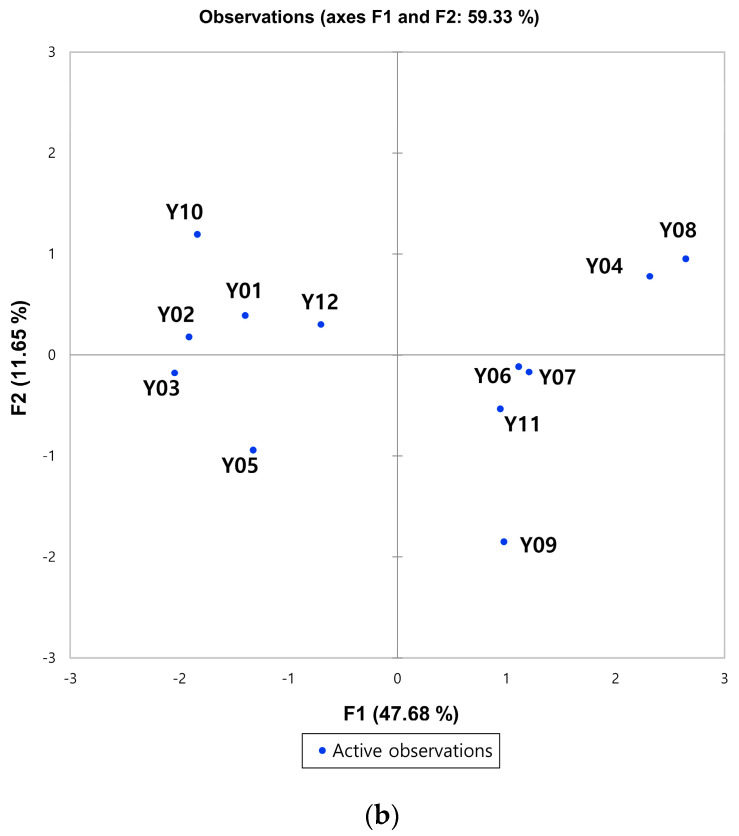
Correlation map of (**a**) descriptive attributes (magenta: O, odor; T, taste; F, flavor; M, mouthfeel; AT, aftertaste; AM, after-mouthfeel), volatile compounds (green) and consumers’ acceptance (red) and (**b**) *yakju* samples in the first two dimension of MFA.

For the MFA, 30 volatile compounds (acids = 3; alcohol = 8; aldehyde = 1; ester = 8; ketone = 2; lactone = 3; phenol = 1; miscellaneous = 4) were selected from the 120 volatile compounds based on a correlation coefficient of >0.5 with consumers’ overall acceptance. A correlation map of descriptive attributes, volatile compounds, and consumer acceptance is in Figure 2a and loading of 12 *yakju* samples in the first two dimensions by MFA is shown in Figure 2b. A total of 59.3% of variance was explained by F1 (47.7%) and F2 (11.7%). Similar to the results in Figure 1, fruit-related sensory attributes were placed on the negative side of F1, while bitterness, mouthfeel, and alcohol flavor were placed on the positive side of F1 (Figure 2a).

Of the volatile compounds, all esters (*n* = 8) were in quadrant 2 and 3, along with fruit-related sensory attributes such as omija odor, hawthorn odor, tangerine peel odor/flavor, apple flavor and grape flavor. These sensory attributes and volatile ester compounds were closely related with consumers’ overall acceptance. Not only two esters (ethyl propanoate, *r* = 0.80; ethyl-2-hydroxy-2-methylbutanoate, *r* = 0.92), but also two lactones (γ-butyrolactone, *r* = 0.85; 4-ethoxycarbonyl-γ-butyrolactone *r* = 0.78), two miscellaneous volatile compounds (2-methyl-1,3-dioxane, *r* = 0.72; 5-hydroxy-2-methyl-1,3-dioxane, *r* = 0.72), and one phenols (4-vinylphenol, *r* = 0.64) were highly correlated with consumers’ overall acceptance (Figure 2a). The volatile esters such as ethyl propanoate and ethyl 2-methyl propanoate in Y1, Y2, Y3, Y5, Y10, and Y12 are known to be found in strawberry juice [37] and durian [38], and methyl 2-furoate is known to be found in dried omija fruit samples [39]. The results of this study implied that the odor characteristics of *yakju* samples might be affected by the volatiles from the fruit or medicinal herbs used in the *yakju* samples. In addition to these volatile esters, ethyl butanoate, known to have a fruity aroma, with an odor threshold of 20 μg/L [40], was positively associated with the overall acceptance scores of most samples (Y1, Y2, Y3, Y5, Y6, Y7, Y,10, Y11, and Y12). However, those of Y4, Y8, and Y9, which were negatively associated with ethyl butanoate, contained under the threshold of ethyl butanoate (Y4 = not detected; Y8 = 16.2 μg/L; Y9 = 7.0 μg/L).

One of the abundant and important volatile lactones found in wine is γ-butyrolactone [25], which has a fruity aroma [31]. Not only γ-butyrolactone but also 4-ethoxycarbonyl-γ-butyrolactone was positively related with consumers’ acceptance. Considering that the content of volatile lactones varies depending on aging time and type of yeast strain in sherry wine [41], the type of yeast strain in the *nuruk* used for the *yakju* samples might have influenced the production of those volatile compounds. In addition, Lee et al. [40] reported that *nuruk* generally contains various kinds of microorganisms compared with to *ipguk* (koji), which only contains *Aspergilus oryzae*, leading to the production of more volatile compounds in *nuruk* than in *ipguk*. Lee et al. [42] reported an absence of 2,3-butanediol in *ipguk* samples, and a relatively low amount of 2,3-butanediol in Y2 (198.9 μg/L) and Y3 (205.5 μg/L) in this study implies that Y2 and Y3 samples might be made of *ipguk.*

Volatile alcohol compounds have a pungent mouthfeel and a pungent and “herbaceous” odor [43]. Described as having a grassy, medicinal, fusel, and spirituous odor, 1-butanol has an odor threshold of 150,000 µg/L [22,43,44]. Although 1-butanol correlated with consumers’ acceptance, the odor of 1-butanol may be imperceptible considering that the 1-butanol contents in the samples ranged from 44.8 μg/L to 1,159.9 μg/L, which was considerably lower than the threshold value.

Volatile phenolic compounds are also formed primarily through alcoholic fermentation [45] and are known to be important in the overall aroma of wine [46] and flavors of dark beer [47]. Generally, volatile phenolic compounds such as 4-vinylphenol have a “nutty” odor similar to “almond shell” [45], spicy, and medicinal-like aromas [48]. Butkhup et al. [46] also reported that these compounds had “heavy pharmaceutical” odor. The existence of 4-vinylphenol was only found in Y1 (111 μg/L), Y2 (73.1 μg/L), Y3 (85.4 μg/L), and Y12 (36.8 μg/L). Although this compound had a positive relationship with young consumers’ acceptance (*r* = 0.64), the effect of 4-vinylphenol on young consumers’ overall acceptance for *yakju* might be negligible considering its threshold (610 μg/L) [46].

Some volatile alcohols (3-butanediol, 2,3-butanediol, 1-octanol, and propylene glycol), acetic acid, γ-hexalactone, and 1,1-diethoxy-3-methylbutane were associated with persimmon vinegar odor, roasted grain odor, yeast odor, kudzu flavor, and coating mouthfeel. Y4 and Y8, which had lower consumer acceptance than other samples, were associated with volatile alcohols such as 1,3-butanediol, 2,3-butanediol, and propylene glycol. This result implied that lower consumer acceptance of these samples might be caused by those volatile alcohol compounds and bitter taste, bitter aftertaste, alcohol flavor, and kudzu flavor. Butanediols were produced from carbohydrates during the alcoholic fermentation primarily by *S. cerevisiae*, a major yeast in *yakju* [49,50,51]. In particular, 2,3-butanediol is the dominant volatile compound in wine, and it has a bitter taste [50]. The highest contents of these compounds in Y4 and Y8 might be due to yeast, such as *S. cerevisiae*, which is involved in the fermentation of rice or supplementary starch ingredients, such as sweet pumpkin and soybean. This result suggested pungent and sour odors like persimmon vinegar odor, related to volatile acetic acid, and some volatile alcohols such as 1,3-butanediol, 2,3-butanediol, and propylene glycol, might negatively affect young consumers’ acceptance.

Furthermore, one of the volatile acetals, 1,1-diethoxy-3-methylbutane, was also associated with Y4 (49.4 μg/L) and Y8 (142 μg/L), which had lower consumers’ acceptance than other samples. This compound was found in liquor samples such as Chinese liquor [23] and Italian grape marc spirit [52]. Volatile acetals are known to be produced by aldehydes in the presence of excessive content of ethanol [23,53]. This suggested that 1,1-diethoxy-3-methylbutane might be produced by relatively high contents of ethanol or aldehyde-related compounds, as in Y4 and Y8. Significant differences in fruit-related attributes were found among the samples, even though the intensities of fruit-related attributes were weak. Thus, overall, the correlation map by MFA showed that fruit-related sensory attributes and volatile compounds were closely related with consumer acceptance. The fruit-related sensory attributes and volatile esters were placed in quadrant 2 and 3, while roasted grain odor, yeast odor, root-related flavor (kudzu flavor), and all mouthfeel attributes were placed in quadrant 1 and 4. The result of this study confirmed the results by Jung et al. [1], who reported that rice wine samples were distinguished by their volatile alcohol and volatile ester. Furthermore, descriptors such as yeast odor and cereal flavor were located opposite to the fruit and sweet aroma in the PCA map.

In addition to volatile compounds that might originate from supplementary ingredients, major ingredients such as different sources of starch might affect the flavors and therefore consumers’ acceptance. While most of the samples were produced mainly from rice, Y1, Y2, Y3, and Y5 used corn starch in addition to rice. Kim et al. [4] conducted a sensory evaluation of traditional liquor samples made from various starch sources. They showed that the acceptance of liquors made with corn starch and brown rice was higher than that of liquors made with glutinous rice or non-glutinous rice or potato starch. Liquors made with corn starch or brown rice tended to contain a relatively lower amount of acetic acid and a higher amount of fructose than the liquors made with non-glutinous rice, suggesting that the type of starch could affect the sensory and chemical properties of liquors. Apart from starch source, rice protein in *yakju* samples increases the pH of *yakju* samples, causing the formation of off-flavor [54]. Moreover, León-Rodríguez et al. [55] suggested that the presence of minor compounds (e.g., some volatile ethyl esters, terpenes, acids, and furans) at low concentrations could cause a synergic effect with other volatile compounds, leading to the production different odor characteristics. Therefore, further investigation on interactions among volatile compounds is needed to understand the odor characteristics and volatile compounds that affect consumers’ overall acceptance.

## 4. Conclusions

Twelve *yakju* samples were characterized, based on sensory descriptors by PCA. The result of the PCA showed that *yakju* samples were characterized mainly by their supplementary raw materials, which affect their overall odor and flavor. As shown by the result of the MFA correlation map, the *yakju* samples tended to be classified by sensory attributes and volatile compounds. The results of this study showed that acceptance of *yakju* samples was largely influenced not only by their alcohol content but also by their supplementary ingredients, especially those related to fruit-related aroma. On the contrary, volatile acetic acid, and some volatile alcohols (1,3-butanediol, 2,3-butanediol, and propylene glycol), and 1,1-diethoxy-3-methylbutane were related with persimmon vinegar odor, roasted grain odor, and yeast odor, and negatively correlated with young consumers’ acceptance of *yakju*. This is a first report on how the major sensory attributes and volatile compounds of Korean rice liquor (*yakju*) affect overall acceptance by young consumers, even though the number of consumers who participated in this study (*n* = 80) was not sufficient. Overall, the results of this study suggested that acceptance of *yakju* products could be improved by controlling some volatile esters that resulted from supplementary ingredients, or specific volatile alcohols and acids produced during fermentation. Considering that the aroma and flavor of *yakju* could vary depending on the starch source, supplementary ingredients, yeast strains, and fermentation process, further investigation is needed on the specific yeast strains and fermentation conditions that affect the formation of volatile compounds and consumers’ acceptance of *yakju*. Along with this, further research on interactions among volatile compounds is also needed to understand the odor characteristics that affect consumers’ overall acceptance.

## Figures and Tables

**Table 1:** Information for the *yakju* samples ^1^.

Sample	Ingredients	Place of Manufacture	Ethanol Content (%)
Y1	Water, rice, starch, high-fructose corn syrup, ginseng, *Schizandra chinensis* fruit (omija), *Poria cocos, Lycium chinense* fruit, *Cornus officinalis, Dioscoreae Rhizoma, Crataegi fructus, Hydrangea macrophylla*, ginger, licorice, *Astragalus propinquus, Acanthopanax sessiliflorus*, yeast, wheat *koji*, citric acid	Gangwon-do, Korea	13.0
Y2	Corn starch, rice, high-fructose corn syrup, sugar, *Crataegi fructus*, lactic acid, *koji*, *Cornus officinalis*, yeast, water	Gyeonggi-do, Korea	13.0
Y3	Water, corn starch, rice, high-fructose corn syrup, sugar, *koji*, lactic acid, orange peel, yeast, dandelion	Gyeonggi-do, Korea	13.0
Y4	Water, rice, sweet pumpkin, *nuruk*	Gangwon-do, Korea	17.0
Y5	Water, starch, glutinous rice, high-fructose corn syrup, wild chrysanthemum, acacia honey, *nuruk*, yeast, purified-yeast, citric acid	Gyeonggi-do, Korea	12.5
Y6	Water, popped rice, rice, glutinous rice, glucose, isomaltooligosaccharide, *nuruk*, balloon flower (root) concentrate, stevioside	Gyeongsang-do, Korea	13.0
Y7	Water, rice, glutinous rice, popped rice, glucose, isomaltooligosaccharide, *nuruk*, balloon flower (root) concentrate, stevioside	Gyeongsang-do, Korea	16.0
Y8	Glutinous rice, rice, *nuruk*, water, wild chrysanthemum, soybean, ginger, red pepper	Chuncheong-do, Korea	18.0
Y9	Water, glutinous/nonglutinous rice, *nuruk*, yeast, (*Dendropanax morbifera* Lev./licorice/*Prunus mume*), *Lentinus edodes* Mycelia, mold starter, refined liquor, aspartame	Jeolla-do, Korea	13.0
Y10	Water, rice, *Schizandra chinensis* fruit (omija), *Cornus officinalis*, *Lycium chinense* fruit, *nuruk*, crude amylolytic enzyme, fructose, sugar, steviol glycoside, glucose, citric acid, malic acid	Jeolla-do, Korea	12.0
Y11	Water, rice, *ipguk (koji)*, *Setaria italica* Beauv., *nuruk*, yeast, refined yeast, licorice, *Sasa borealis*, *Artemisia apiacea* Hance, high-fructose corn syrup	Jeju, Korea	15.0
Y12	Rice, *nuruk*, dried orange peel, yeast, crude amylolytic enzyme, purified yeast, high-fructose corn syrup, citric acid, steviol glycoside	Jeju, Korea	11.0

^1^ Information on ingredients of products was obtained from the label of each product.

**Table 2:** Descriptors, definitions, and reference materials of 12 *yakju* samples.

Attributes	Definition	Reference Materials	Reference Intensity
**Appearance**			
Clearness	Degree of turbidity	Water	15.0
Redness	Degree of redness	Pantone color book (Pantone, NJ, USA)	487 C = 7.5; 485 C = 15.0
Yellowness	Degree of yellowness	Pantone color book (Pantone, NJ, USA)	129 C = 7.5; 131 C = 15.0
**Odor**			
Alcohol	Odor associated with alcohol	*Soju* (Chamisul Fresh, Hitejinro Co., Ltd., Seoul, Korea)	8.44
Chrysanthemum	Aroma associated with chrysanthemum	Chrysanthemum teabag (PurunSan Agricultural Co., Seoul, Korea)	14.5
Ginseng	Aroma associated with ginseng	Ginseng powder (PurunSan Agricultural Co., Seoul, Korea)	14.5
Grape	Aroma associated with grape	Grape	14.5
Hawthorn (*Crataegi fructus)*	Aroma associated with hawthorn fruit (*Crataegi fructus)*	Dried hawthorn fruit (PurunSan Agricultural Co., Seoul, Korea)	14.5
Maesil (*Prunus mume* fruit)	Maesil aroma	Maesil	14.5
*Makgeolli*	Sour and sweet aroma associated with *Nuruk*, *ipguk (koji)*, and fermentation.	Rice *Makgeolli* (Kooksoondang Brewery Co., Ltd., Gangwon-do, Korea)	10.9
Omija (*Schizandra chinensis* fruit)	Aroma associated with omija	omija	14.5
Peppermint	Aroma associated with peppermint	Peppermint (Lipton peppermint herb tea, Unilever, London, England)	14.5
Persimmon vinegar	Sweet and acidic aroma associated with persimmon vinegar	Persimmon vinegar (Chungjungone Co., Seoul, Korea)	13.3
Pine	Aroma associated with pine	Pine Bud Drink (Lotte Co., Ltd., Seoul, Korea)	13.6
Roasted grain	Savory aroma associated with roasted grain	Cornsilk Tea Drink (Kwangdong Pharmaceutical Co., Ltd., Seoul, Korea	11.7
Sour	Sour aroma associated with vinegar, fruit	Vinegar solution (Brewed vinegar, Ottogi, Gyeonggi-do, Korea)	Vinegar vs. water (1:1) = 7.60; (2:1) = 11.4
Sweet	Sweet aroma associated with honey, syrup	Rice syrup (Chungjungone Co., Seoul, Korea)	10.0
Tangerine peel	Aroma associated with tangerine peel	Tangerine peel powder (PurunSan Agricultural Co., Seoul, Korea)	14.5
Yeast	Salty and moldy aroma associated with *meju*, soy sauce, and soybean paste	Yeast (Jeonwon Foods Co., Gyeonggi-do, Korea)	12.2
**Taste/Flavor**			
Bitterness	Fundamental taste of bitterness	1.0% (*w*/*w*) guarana solution (Guarana extract powder, Cremar, Seoul, Korea)	5.3
Saltiness	Fundamental taste of saltiness	0.1% (*w*/*w*) NaCl solution (Morton iodized salt, Morton Salt, Inc., Chicago, IL, USA)	2.5
Sourness	Fundamental taste of sourness	0.1% (*w*/*w*) citric acid solution (EdentownF&B, Incheon, Korea)	5.5
Sweetness	Fundamental taste of sweetness	1.0% (*w*/*w*) sucrose solution (CJ Cheiljedang Co., Seoul, Korea)	2.40
Umami	Fundamental taste of umami	0.5% MSG solution (Miwon, Daesang Co., Seoul, Korea)	13.0
Alcohol	Flavor associated with alcohol	*Soju* (Chamisul Fresh, Hitejinro Co., Ltd., Seoul, Korea)	7.50
Apple	Flavor associated with apple	Apple	14.5
Balloon flower root	Flavor associated with balloon flower root	Balloon flower root	14.5
Grape	Flavor associated with grape	Grape	14.5
Maesil *(Prunus mume* fruit)	Flavor associated with maesil	Maesil	14.5
Roasted grain	Savory flavor associated with roasted grain	Cornsilk Tea Drink (Kwangdong Pharmaceutical Co., Ltd., Seoul, Korea)	13.1
Tangerine peel	Flavor associated with tangerine peel	Tangerine peel powder (PurunSan Agricultural Co., Seoul, Korea)	14.5
**Mouthfeel**			
Astringent	Dry and a feeling of shrank skin in the mouth	0.3% (*w*/*w*) alum solution (Alum, McCormick & Co., Inc., Baltimore, MD, USA)	2.6
Body	Mouthfeel associated richness and heaviness	*Soju* (Chamisul Fresh, Hitejinro Co., Ltd., Seoul, Korea)	4.5
Burning	Mouthfeel associated with mouth-burning feeling caused by alcohol	*Soju* (Chamisul Fresh, Hitejinro Co., Ltd., Seoul, Korea)	8.7
Coating	Mouthfeel associated with wrapping the mouth with a soft and slippery feeling	*Soju* (Chamisul Fresh, Hitejinro Co., Ltd., Seoul, Korea)	4.6
Pungent	Mouthfeel associated with stimulation of the nasal cavity and mouth	Persimmon vinegar (Chungjungone Co., Seoul, Korea)	14.5
Stinging	Mouthfeel associated with stinging, tingling sensation	Radish sprouts	13.5
**Aftertaste/mouthfeel**			
Bitterness	Taste of bitterness after swallowing	1.0% (*w*/*w*) Guarana solution (Guarana extract powder, Cremar, Seoul, Korea)	5.26
Sourness	Taste of sourness after swallowing	0.1% (*w*/*w*) citric acid solution (EdentownF&B, Incheon, Korea)	5.47
Sweetness	Taste of sweetness after swallowing	1.0% (*w*/*w*) sucrose solution (CJ Cheiljedang Co., Seoul, Korea)	2.40
Umami	Taste of umami after swallowing	0.5% MSG solution (Miwon, Daesang Co., Seoul, Korea)	13.0
Astringent	Mouthfeel of drying, shrinking after swallowing	0.3% (*w*/*w*) alum solution (Alum, McCormick & Co., Inc., Baltimore, MD, USA)	2.6
Burning	Mouthfeel of alcohol after swallowing	*Soju* (Chamisul Fresh, Hitejinro Co., Ltd., Seoul, Korea)	8.7
Coating	Mouthfeel of wrapping the mouth with a soft and slippery feeling after swallowing	*Soju* (Chamisul Fresh, Hitejinro Co., Ltd., Seoul, Korea)	4.6
Residue	Mouthfeel of residue after swallowing	Milk (SeoulMilk, Seoul, Korea)	9.60
Stinging	Mouthfeel of stinging, tingling sensation after swallowing	*Soju* (Chamisul Fresh, Hitejinro Co., Ltd., Seoul, Korea)	13.5

**Table 3:** Mean intensity scores of the sensory attributes for 12 *yakju* samples ^1–3^.

	Samples	Y1	Y2	Y3	Y4	Y5	Y6	Y7	Y8	Y9	Y10	Y11	Y12
Attributes													
**Appearance**													
Clearness		14.4 ± 0.95	14.4 ± 0.94	14.6 ± 0.57	14.2 ± 1.39	14.3 ± 2.59	14.2 ± 1.29	14.2 ± 1.37	13.8 ± 1.81	14.3 ± 2.60	14.0 ± 1.87	14.6 ± 0.64	14.4 ± 0.92
Redness ***		1.25 ^c^ ± 1.39	2.07 ^b^ ± 2.25	0.00 ^d^ ± 0.02	0.03 ^d^ ± 0.08	0.43 ^d^ ± 2.45	0.04 ^d^ ± 0.11	0.03 ^d^ ± 0.09	0.08 ^d^ ± 0.22	0.02 ^d^ ± 0.09	11.5 ^a^ ± 2.72	0.01 ^d^ ± 0.03	0.00 ^d^ ± 0.01
Yellowness ***		4.20 ^c^ ± 3.50	2.98 ^d^ ± 3.41	2.02 ^d^ ± 1.67	6.95 ^b^ ± 2.90	1.40 ^d^ ± 0.71	7.58 ^b^ ± 2.46	9.18 ^a^ ± 2.30	10.1 ^a^ ± 1.99	1.37 ^d^ ± 0.88	1.71 ^d^ ± 4.00	1.98 ^d^ ± 1.41	4.67 ^c^ ± 2.06
**Odor**													
Apple **		0.21 ^ab^ ± 0.56	0.43 ^ab^ ± 1.06	0.46 ^ab^ ± 0.74	0.22 ^ab^ ± 0.45	0.12 ^b^ ± 0.34	0.12 ^b^ ± 0.38	0.03 ^b^ ± 0.09	0.19 ^ab^ ± 0.65	0.29 ^ab^ ± 0.53	0.32 ^ab^ ± 0.91	0.71 ^a^ ± 1.24	0.19 ± 0.51
Alcohol		3.65 ± 2.03	3.53 ± 1.83	3.75 ± 2.06	3.71 ± 1.87	3.50 ± 1.95	3.83 ± 1.91	3.81 ± 1.67	3.56 ± 2.14	3.78 ± 1.99	3.48 ± 1.94	3.56 ± 2.19	3.20 ± 1.84
Chrysanthemum ***		0.09 ^b^ ± 0.41	0.03 ^b^ ± 0.11	0.02 ^b^ ± 0.09	0.05 ^b^ ± 0.18	2.09 ^a^ ± 2.98	0.19 ^b^ ± 0.46	0.15 ^b^ ± 0.34	0.16 ^b^ ± 0.51	0.02 ^b^ ± 0.12	0.60 ^b^ ± 2.66	0.26 ^b^ ± 1.19	0.64 ^b^ ± 1.45
Ginseng ***		1.97 ^a^ ± 2.41	0.06 ^b^ ± 0.22	0.46 ^b^ ± 1.78	0.21 ^b^ ± 0.83	0.09 ^b^ ± 0.37	0.06 ^b^ ± 0.18	0.39 ^b^ ± 1.47	0.53 ^b^ ± 1.67	0.40 ^b^ ± 1.12	0.11 ^b^ ± 0.57	0.34 ^b^ ± 1.22	0.03 ^b^ ± 0.08
Hawthorn ***		1.38 ^a^ ± 2.11	0.80 ^abc^ ± 1.57	0.29 ^bc^ ± 1.21	0.24 ^bc^ ± 0.56	0.48 ^bc^ ± 1.15	0.22 ^bc^ ± 0.45	0.19 ^bc^ ± 0.36	0.10 ^bc^ ± 0.22	0.01 ^c^ ± 0.03	0.86 ^ab^ ± 0.87	0.02 ^c^ ± 0.06	0.67 ^bc^ ± 1.32
Maesil (*Prunus mume* fruit)		0.74 ± 1.27	1.22 ± 2.12	0.63 ± 0.81	0.82 ± 1.14	0.31 ± 0.55	0.77 ± 0.85	0.66 ± 1.06	0.88 ± 1.21	0.51 ± 0.88	0.75 ± 1.39	0.64 ± 0.80	0.59 ± 0.95
*Makgeolli*		2.22 ± 1.81	2.21 ± 1.73	2.70 ± 1.96	2.26 ± 1.41	1.89 ± 2.02	2.57 ± 1.63	2.45 ± 2.17	1.96 ± 1.33	2.49 ± 1.75	1.32 ± 1.43	2.69 ± 1.97	1.63 ± 1.35
Omija (*Schizandra chinensis* fruit) ***		0.98 ^b^ ± 1.49	0.50 ^b^ ± 0.87	0.01 ^b^ ± 0.02	0.27 ^b^ ± 0.94	0.17 ^b^ ± 0.38	0.17 ^b^ ± 0.50	0.13 ^b^ ± 0.32	0.16 ^b^ ± 0.48	0.00 ^b^ ± 0.02	5.75 ^a^ ± 3.63	0.01 ^b^ ± 0.02	0.28 ^b^ ± 0.64
Peppermint ***		0.13 ^b^ ± 0.50	0.07 ^b^ ± 0.41	0.05 ^b^ ± 0.17	0.26 ^b^ ± 1.04	1.11 ^a^ ± 1.86	0.07 ^b^ ± 0.38	0.00 ^b^ ± 0.02	0.07 ^b^ ± 0.41	0.00 ^b^ ± 0.01	0.17 ^b^ ± 0.64	0.00 ^b^ ± 0.02	0.47 ^b^ ± 1.02
Persimmon Vinegar ***		0.34 ^bcd^ ± 0.81	0.36 ^bcd^ ± 0.64	0.11 ^d^ ± 0.24	1.09 ^a^ ± 1.65	0.06 ^d^ ± 0.17	0.98 ^ab^ ± 1.25	0.74 ^abcd^ ± 1.11	0.86 ^abc^ ± 1.33	0.24 ^cd^ ± 0.44	0.64 ^abcd^ ± 1.34	0.31 ^bcd^ ± 0.64	0.21 ^cd^ ± 0.37
Pine ***		0.10 ^b^ ± 0.28	0.01 ^b^ ± 0.02	0.06 ^b^ ± 0.23	0.14 ^b^ ± 0.81	2.05 ^a^ ± 2.49	0.00 ^b^ ± 0.01	0.01 ^b^ ± 0.02	0.05 ^b^ ± 0.27	0.00 ^b^ ± 0.02	0.10 ^b^ ± 0.47	0.01 ^b^ ± 0.02	0.71 ^b^ ± 1.51
Pineapple **		0.02 ^b^ ± 0.06	0.14 ^b^ ± 0.35	0.30 ^b^ ± 1.31	0.20 ^b^ ± 0.89	0.15 ^b^ ± 0.50	0.27 ^b^ ± 0.81	0.01 ^b^ ± 0.03	0.02 ^b^ ± 0.09	0.11 ^b^ ± 0.22	0.14 ^b^ ± 0.58	1.02 ^a^ ± 2.63	0.07 ^b^ ± 0.34
Roasted grain ***		0.72 ^ab^ ± 1.37	0.49 ^b^ ± 0.82	0.93 ^ab^ ± 1.21	1.32 ^ab^ ± 2.14	0.30 ^b^ ± 0.59	1.53 ^a^ ± 2.20	1.15 ^ab^ ± 1.60	1.60 ^a^ ± 2.01	0.43 ^b^ ± 0.61	0.78 ^ab^ ± 1.15	0.41 ^b^ ± 0.55	0.43 ^b^ ± 0.58
Sour		2.94 ± 2.87	3.11 ± 2.76	2.70 ± 2.41	3.67 ± 3.00	2.69 ± 2.59	3.00 ± 2.70	2.64 ± 2.57	3.60 ± 3.35	2.36 ± 2.53	4.15 ± 3.09	3.04 ± 2.70	3.01 ± 3.02
Sweet		2.93 ± 1.84	2.65 ± 1.88	2.42 ± 1.47	2.99 ± 1.58	2.40 ± 1.98	3.05 ± 1.83	2.58 ± 1.68	3.28 ± 1.54	2.58 ± 1.51	3.68 ± 2.30	3.06 ± 2.03	2.94 ± 1.52
Tangerine peel ***		0.25 ^b^ ± 0.67	0.08 ^b^ ± 0.25	0.22 ^b^ ± 0.96	0.37 ^b^ ± 0.82	0.19 ^b^ ± 0.38	0.11 ^b^ ± 0.22	0.40 ^b^ ± 0.86	0.20 ^b^ ± 0.43	0.07 ^b^ ± 0.23	0.33 ^b^ ± 0.94	0.09 ^b^ ± 0.36	1.13 ^a^ ± 1.32
Yeast ***		0.89 ^b^ ± 1.38	1.86 ^b^ ± 2.10	0.91 ^b^ ± 1.00	3.26 ^a^ ± 2.05	0.65 ^b^ ± 0.88	3.60 ^a^ ± 2.70	3.73 ^a^ ± 2.55	3.46 ^a^ ± 2.27	1.00 ^b^ ± 1.22	0.86 ^b^ ± 1.25	1.68 ^b^ ± 1.98	1.25 ^b^ ± 1.18
**Taste/Flavor**													
Bitterness **		2.59 ^abc^ ± 1.96	1.77 ^c^ ± 1.54	2.19 ^abc^ ± 1.57	2.96 ^abc^ ± 2.18	2.65 ^abc^ ± 1.67	2.78 ^abc^ ± 1.48	3.55 ^a^ ± 1.96	3.24 ^ab^ ± 2.10	3.05 ^abc^ ± 1.81	2.40 ^abc^ ± 1.48	2.60 ^abc^ ± 1.87	2.12 ^bc^ ± 1.45
Saltiness ***		0.85 ^bc^ ± 0.90	0.85 ^bc^ ± 0.93	0.85 ^bc^ ± 1.00	1.52 ^ab^ ± 1.19	0.53 ^c^ ± 0.72	1.35 ^ab^ ± 1.17	1.37 ^ab^ ± 1.19	1.89 ^a^ ± 1.42	0.48 ^c^ ± 0.62	1.23 ^abc^ ± 1.07	0.90 ^bc^ ± 0.93	1.04 ^bc^ ± 0.93
Sourness ***		3.26 ^bcd^ ± 1.93	3.81 ^abc^ ± 2.25	2.86 ^bcd^ ± 1.87	4.74 ^a^ ± 2.07	2.39 ^d^ ± 1.66	2.97 ^bcd^ ± 1.67	2.97 ^bcd^ ± 1.84	2.62 ^cd^ ± 2.23	2.33 ^d^ ± 2.08	4.23 ^ab^ ± 1.60	4.07 ^ab^ ± 1.80	2.88 ^bcd^ ± 1.46
Sweetness ***		2.80 ^abc^ ± 1.57	3.22 ^a^ ± 1.75	3.03 ^ab^ ± 1.56	2.67 ^abc^ ± 1.94	2.60 ^abc^ ± 1.47	1.85 ^cd^ ± 1.16	1.93 ^bcd^ ± 1.38	3.06 ^ab^ ± 1.58	1.36 ^d^ ± 0.89	3.37 ^a^ ± 1.73	2.59 ^abc^ ± 1.86	2.83 ^abc^ ± 1.40
Umami ***		0.83 ^b^ ± 1.08	0.77 ^b^ ± 1.04	0.81 ^b^ ± 0.94	1.27 ^ab^ ± 1.71	0.71 ^b^ ± 1.19	1.21 ^ab^ ± 1.94	1.03 ^b^ ± 1.69	2.14 ^a^ ± 2.20	0.50 ^b^ ± 1.10	1.04 ^b^ ± 1.43	0.70 ^b^ ± 0.93	1.48 ^ab^ ± 1.58
Alcohol ***		3.78 ^ab^ ± 1.82	3.56 ^ab^ ± 1.76	3.55 ^ab^ ± 1.77	4.49 ^ab^ ± 2.21	3.89 ^ab^ ± 1.78	4.56 ^ab^ ± 1.55	4.86 ^a^ ± 2.01	4.79 ^a^ ± 1.77	4.76 ^a^ ± 1.84	3.25 ^b^ ± 1.40	4.13 ^ab^ ± 1.90	3.27 ^b^ ± 1.69
Apple **		0.64 ^ab^ ± 1.42	0.89 ^a^ ± 1.29	0.81 ^ab^ ± 1.50	0.38 ^ab^ ± 1.06	0.40 ^ab^ ± 0.88	0.13 ^ab^ ± 0.28	0.03 ^b^ ± 0.09	0.03 ^b^ ± 0.07	0.13 ^ab^ ± 0.37	0.59 ^ab^ ± 1.21	0.66 ^ab^ ± 1.33	0.54 ^ab^ ± 1.24
Balloon flower root		1.10 ± 1.67	0.23 ± 0.54	0.58 ± 1.94	0.60 ± 1.56	0.84 ± 1.38	0.70 ± 1.19	0.69 ± 1.21	0.74 ± 1.69	1.12 ± 2.13	0.33 ± 0.64	0.61 ± 1.48	0.33 ± 0.60
Grape ***		0.73 ^ab^ ± 1.40	0.87 ^a^ ± 1.44	0.61 ^ab^ ± 1.50	0.33 ^ab^ ± 0.95	0.31 ^ab^ ± 0.88	0.09 ^b^ ± 0.31	0.01 ^b^ ± 0.03	0.02 ^b^ ± 0.09	0.05 ^b^ ± 0.14	0.89 ^a^ ± 1.39	0.79 ^ab^ ± 1.53	0.26 ^ab^ ± 0.80
Kudzu *		0.23 ^ab^ ± 0.44	0.03 ^b^ ± 0.07	0.37 ^ab^ ± 1.40	0.51 ^ab^ ± 1.17	0.40 ^ab^ ± 0.81	0.54 ^ab^ ± 1.27	0.88 ^a^ ± 1.92	0.68 ^ab^ ± 1.50	0.30 ^ab^ ± 0.67	0.08 ^ab^ ± 0.26	0.15 ^ab^ ± 0.63	0.19 ^ab^ ± 0.39
Maesil (*Prunus mume* fruit)		0.99 ± 2.18	1.05 ± 1.72	0.78 ± 1.54	0.91 ± 1.57	0.60 ± 1.07	0.29 ± 0.55	0.17 ± 0.36	0.45 ± 0.62	0.23 ± 0.47	1.11 ± 1.63	0.64 ± 1.10	0.78 ± 1.45
Roasted grain		1.23 ± 1.77	0.83 ± 1.35	1.36 ± 1.65	1.57 ± 1.97	0.67 ± 0.83	1.84 ± 2.51	0.97 ± 1.20	1.97 ± 2.26	0.79 ± 1.31	1.26 ± 1.54	0.86 ± 1.04	1.09 ± 1.17
Tangerine peel **		0.40 ^b^ ± 0.88	0.18 ^b^ ± 0.42	0.27 ^b^ ± 0.90	0.55 ^b^ ± 0.88	0.51 ^b^ ± 0.73	0.42 ^b^ ± 1.00	0.53 ^b^ ± 1.28	0.20 ^b^ ± 0.39	0.33 ^b^ ± 0.96	0.61 ^b^ ± 1.03	0.47 ^b^ ± 1.20	1.22 ^a^ ± 1.44
**Mouthfeel**													
Astringent		1.01 ± 0.82	1.16 ± 0.80	1.12 ± 0.89	1.26 ± 1.17	0.82 ± 0.74	1.05 ± 0.91	1.13 ± 0.81	0.93 ± 0.83	0.99 ± 1.09	1.24 ± 0.67	1.15 ± 0.85	0.83 ± 0.63
Body ***		3.04 ^abc^ ± 2.12	2.82 ^bc^ ± 2.21	2.14 ^c^ ± 1.69	3.82 ^ab^ ± 2.36	2.23 ^c^ ± 1.79	3.08 ^abc^ ± 1.95	3.15 ^abc^ ± 1.92	4.43 ^a^ ± 2.48	1.98 ^c^ ± 1.43	3.32 ^abc^ ± 1.97	2.56 ^bc^ ± 2.10	3.11 ^abc^ ± 1.91
Burning		3.56 ± 2.65	3.25 ± 2.74	3.62 ± 2.74	4.67 ± 3.01	3.54 ± 2.58	4.00 ± 2.53	4.80 ± 2.80	4.84 ± 2.95	4.77 ± 2.60	2.91 ± 2.30	4.21 ± 2.76	3.14 ± 2.47
Coating **		1.84 ^b^ ± 1.42	1.72 ^b^ ± 1.48	1.60 ^b^ ± 1.33	2.64 ^ab^ ± 1.71	1.66 ^b^ ± 1.14	2.00 ^ab^ ± 1.31	2.14 ^ab^ ± 1.46	2.92 ^a^ ± 1.94	2.02 ^ab^ ± 1.35	1.67 ^b^ ± 1.24	1.83 ^b^ ± 1.33	1.77 ^b^ ± 1.44
Pungent		0.87 ± 1.07	0.75 ± 1.16	0.65 ± 0.98	1.67 ± 2.16	0.70 ± 0.82	1.08 ± 1.51	1.36 ± 2.34	1.15 ± 1.45	1.11 ± 1.44	1.21 ± 1.85	1.02 ± 1.34	0.84 ± 1.19
Stinging ***		1.54 ^b^ ± 1.48	1.26 ^b^ ± 1.22	1.48 ^b^ ± 1.48	3.03 ^a^ ± 2.69	1.77 ^ab^ ± 1.69	2.31 ^ab^ ± 2.01	2.62 ^ab^ ± 2.00	2.70 ^ab^ ± 2.53	2.42 ^ab^ ± 2.19	1.43 ^b^ ± 1.42	1.87 ^ab^ ± 1.64	1.49 ^b^ ± 1.40
**Aftertaste/mouthfeel**													
Bitterness **		1.75 ^ab^ ± 1.70	1.25 ^b^ ± 1.37	1.65 ^ab^ ± 1.47	2.21 ^ab^ ± 2.06	1.86 ^ab^ ± 1.32	2.11 ^ab^ ± 1.19	2.72 ^a^ ± 1.80	2.29 ^ab^ ± 1.80	2.18 ^ab^ ± 1.76	1.53 ^ab^ ± 1.03	2.00 ^ab^ ± 1.61	1.43 ^b^ ± 1.23
Sourness ***		2.16 ^bc^ ± 1.62	2.38 ^bc^ ± 1.72	1.77 ^bc^ ± 1.23	3.44 ^a^ ± 1.78	1.41 ^c^ ± 1.00	2.06 ^bc^ ± 1.46	2.19 ^bc^ ± 1.55	1.78 ^bc^ ± 1.65	1.38 ^c^ ± 1.45	2.72 ^ab^ ± 1.43	2.68 ^ab^ ± 1.54	1.91 ^bc^ ± 1.08
Sweetness ***		1.78 ^abc^ ± 0.65	2.04 ^ab^ ± 0.84	1.93 ^ab^ ± 0.70	1.63 ^bcd^ ± 0.78	1.40 ^cd^ ± 0.56	1.23 ^de^ ± 0.63	1.18 ^de^ ± 0.67	2.16 ^a^ ± 0.80	0.84 ^e^ ± 0.60	1.94 ^ab^ ± 0.95	1.41 ^cd^ ± 0.67	1.84 ^abc^ ± 0.75
Umami **		0.81 ^abc^ ± 0.99	0.59 ^abc^ ± 0.50	0.79 ^abc^ ± 1.11	1.05 ^abc^ ± 1.45	0.46 ^bc^ ± 0.81	0.83 ^abc^ ± 1.36	0.75 ^abc^ ± 1.15	1.36 ^a^ ± 0.91	0.23 ^c^ ± 0.31	0.97 ^abc^ ± 1.35	0.52 ^bc^ ± 0.87	1.09 ^ab^ ± 1.15
Astringent		0.79 ± 0.88	0.88 ± 0.73	0.81 ± 0.77	0.95 ± 0.95	0.84 ± 0.74	0.87 ± 0.69	1.07 ± 1.12	0.83 ± 0.76	0.76 ± 0.98	1.05 ± 0.70	1.01 ± 0.89	0.68 ± 0.50
Burning		2.74 ± 2.35	2.18 ± 2.08	2.67 ± 2.05	3.39 ± 2.24	2.40 ± 2.17	3.15 ± 2.28	3.34 ± 2.03	3.67 ± 2.56	3.60 ± 2.42	1.91 ± 1.46	3.10 ± 2.17	2.31 ± 2.04
Coating		1.22 ± 0.91	1.15 ± 1.01	1.26 ± 1.03	1.70 ± 1.27	1.29 ± 1.04	1.39 ± 0.90	1.52 ± 1.06	1.86 ± 1.23	1.55 ± 0.95	1.08 ± 0.89	1.35 ± 1.07	1.33 ± 1.02
Residue **		0.92 ^ab^ ± 0.92	0.92 ^ab^ ± 0.84	0.81 ^ab^ ± 0.74	1.47 ^ab^ ± 1.26	0.83 ^ab^ ± 0.78	1.33 ^ab^ ± 1.55	1.09 ^ab^ ± 1.00	1.58 ^a^ ± 1.09	0.72 ^b^ ± 0.67	1.21 ^ab^ ± 1.21	0.80 ^ab^ ± 0.70	1.04 ^ab^ ± 0.96
Stinging ***		1.30 ^abc^ ± 1.49	0.86 ^c^ ± 0.79	1.06 ^bc^ ± 1.00	2.32 ^a^ ± 2.44	1.32 ^abc^ ± 1.50	1.74 ^abc^ ± 1.88	2.07 ^abc^ ± 1.58	2.16 ^ab^ ± 1.96	1.97 ^abc^ ± 1.76	0.88 ^c^ ± 0.90	1.33 ^abc^ ± 1.54	1.19 ^abc^ ± 1.13

^1^ Mean values with different alphabet mean significantly different. ^2^ *^,^ **^,^ *** means significantly different at *p* < 0.05, *p* < 0.01, and *p* < 0.001 respectively. ^3^ 15 cm line scale was used; 0 cm = none, 15 cm = very stron.

**Table 4:** Mean scores for the consumers’ acceptance of the *yakju* samples ^1,2^.

Samples	Overall Acceptance Scores
Y1	5.12 ^d^ ± 2.10
Y2	6.34 ^ab^ ± 1.82
Y3	6.71 ^a^ ± 1.34
Y4	4.01 ^e^ ± 2.13
Y5	5.59 ^cd^ ± 1.80
Y6	4.02 ^e^ ± 1.71
Y7	3.43 ^f^ ± 1.57
Y8	3.25 ^f^ ± 2.25
Y9	4.06 ^e^ ± 1.72
Y10	6.41 ^ab^ ± 1.76
Y11	4.30 ^e^ ± 1.66
Y12	5.82 ^bc^ ± 1.63

^1^ Average scores of 80 consumers; 1 = dislike extremely, 9 = like extremely. ^2^ Mean values with different alphabet mean significantly different at *p* < 0.05.

**Table 5:** Volatile compounds of the 12 *yakju* samples ^1^.

RI ^2^	Compound	Concentration (μg/L)											
		Y1	Y2	Y3	Y4	Y5	Y6	Y7	Y8	Y9	Y10	Y11	Y12
	***Acids***												
1434	acetic acid	1314 ± 319	837 ± 176	414 ± 60.8	14,700 ± 1524	894 ± 117	1230 ± 330	1281 ± 447	5038 ± 301	1558 ± 371	1436 ± 185	2266 ± 407	624 ± 69.8
1492	formic acid	ND	ND	ND	ND	ND	ND	11.3 ± 6.7	ND	ND	ND	ND	ND
1524	propanoic acid	43.0 ± 7.06	41.7 ± 10.0	32.6 ± 3.78	751 ± 115	843 ± 95.1	72.6 ± 7.00	90.7 ± 17.0	65.3 ± 4.60	200 ± 38.4	76.9 ± 7.36	113 ± 12.6	78.5 ± 5.50
1554	2-methyl propanoic acid	479 ± 85.3	494 ± 80.5	453 ± 50.0	187 ± 59.4	662 ± 82.6	110 ± 21.0	138 ± 24.9	309 ± 20.6	105 ± 13.6	465 ± 48.4	451 ± 70.3	313 ± 18.8
1613	butanoic acid	148 ± 25.3	172 ± 27.8	137 ± 16.4	84.4 ± 27.9	140 ± 19.9	104 ± 23.3	114 ± 18.9	207 ± 14.3	20.1 ± 2.82	146 ± 16.0	178 ± 27.0	162 ± 11.9
1656	3-methyl butanoic acid	422 ± 61.5	385 ± 73.7	303 ± 27.1	254 ± 67.2	540 ± 59.8	143 ± 52.2	145 ± 16.9	330 ± 28.2	104 ± 8.31	324 ± 49.7	399 ± 21.2	226 ± 45.2
1724	pentanoic acid	ND	16.2 ± 0.69	ND	22.3 ± 9.64	ND	43.2 ± 6.05	56.6 ± 1.40	44.2 ± 8.33	ND	73.1 ± 5.70	ND	29.1 ± 6.62
1759	2-butenoic acid	ND	ND	ND	ND	ND	ND	12.4 ± 1.79	ND	ND	ND	ND	ND
1771	2-methyl-2-butenoic acid	33.2 ± 7.04	26.1 ± 8.91	33.2 ± 14.3	ND	35.2 ± 10.3	25.9 ± 6.13	39.2 ± 11.4	ND	10.9 ± 0.86	76.3 ± 32.1	51.0 ± 32.2	17.7 ± 5.12
1832	hexanoic acid	189 ± 28.7	232 ± 35.3	182 ± 18.5	301 ± 151	308 ± 37.4	168 ± 7.83	206 ± 21.6	282 ± 22.2	56.7 ± 6.48	194 ± 19.7	232 ± 48.7	304 ± 34.5
2034	octanoic acid	88.1 ± 9.82	144 ± 24.4	122 ± 4.65	113 ± 78.7	89.6 ± 16.8	142 ± 2.54	155 ± 31.0	60.3 ± 21.3	112 ± 17.9	122 ± 15.4	171 ± 27.8	293 ± 16.7
2170	lactic acid	350 ± 131	1777 ± 421	1018 ± 149	2277 ± 1032	211 ± 135	215 ± 62.7	81.8 ± 48.2	73.3 ± 91.7	ND	80.3 ± 16.6	ND	ND
2256	decanoic acid	19.4 ± 9.54	19.9 ± 4.46	15.8 ± 2.74	33.5 ± 16.5	14.7 ± 1.26	ND	ND	ND	ND	ND	ND	30.6 ± 6.40
2422	benzoic acid	36.6 ± 5.43	24.2 ± 7.04	26.2 ± 5.82	46.7 ± 7.00	38.3 ± 8.53	16.2 ± 4.79	39.5 ± 12.1	19.6 ± 3.92	27.6 ± 8.33	31.0 ± 5.63	17.8 ± 1.53	57.0 ± 9.00
	**Subtotal**	3122 ± 362	4170 ± 473	2737 ± 174	18,770 ± 1855	3776 ± 232	2269 ± 342	2372 ± 453	6429 ± 319	2194 ± 374	3024 ± 204	3879 ± 420	2135 ± 95.4
	***Alcohols***												
1025	1-propanol	777 ± 142	727 ± 125	735 ± 68.1	510 ± 106	663 ± 37.7	814 ± 161	985 ± 101	500 ± 42.7	487 ± 86.9	963 ± 110	1969 ± 155	954 ± 95.0
1085	2-methyl-1-propanol	10,085 ± 1526	12,313 ± 1612	9109 ± 517	4251 ± 1018	10,221 ± 919	6989 ± 1035	7703 ± 631	9469 ± 706	4303 ± 558	7171 ± 815	7366 ± 754	8548 ± 886
1134	1-butanol	247 ± 28.8	300 ± 52.4	735 ± 43.4	44.8 ± 9.4	132 ± 11.4	456 ± 75.5	460 ± 48.8	119 ± 6.45	288 ± 41.2	1160 ± 107	289 ± 36.0	511 ± 77.8
1210	isoamyl alcohol	41,034 ± 5751	44,956 ± 5725	39,224 ± 1480	15,567 ± 8214	45,159 ± 4540	31,676 ± 5269	36,373 ± 2475	34,114 ± 1974	18,342 ± 2092	32,551 ± 2998	35,548 ± 4850	30,988 ± 1902
1241	3-methyl-3-buten-1-ol	43.3 ± 4.29	30.0 ± 6.04	28.9 ± 2.88	43.7 ± 20.1	75.3 ± 3.78	49.6 ± 3.62	67.2 ± 15.8	70.9 ± 2.74	14.8 ± 2.45	48.8 ± 2.22	53.9 ± 10.58	25.5 ± 2.20
1248	1-pentanol	ND	ND	ND	ND	ND	ND	ND	16.7 ± 2.49	ND	17.5 ± 6.76	ND	ND
1311	2-methyl-2-buten-1-ol	17.0 ± 2.28	ND	ND	50.7 ± 15.1	ND	ND	14.2 ± 3.21	10.6 ± 0.61	ND	10.5 ± 0.69	24.5 ± 5.31	25.6 ± 1.86
1322	3-methyl-1-pentanol	12.0 ± 4.04	18.2 ± 2.01	16.6 ± 1.19	ND	16.5 ± 1.57	8.91 ± 0.21	12.4 ± 0.78	8.01 ± 1.09	ND	14.2 ± 1.71	12.6 ± 8.13	8.59 ± 0.41
1342	1-hexanol	71.8 ± 11.1	ND	ND	ND	171 ± 16.3	111 ± 11.6	169 ± 16.1	164 ± 7.98	17.2 ± 1.52	34.0 ± 7.23	36.6 ± 6.48	20.6 ± 1.37
1370	3-ethoxy-1-propanol	1684. ± 296	507 ± 78.2	868 ± 86.6	248 ± 89.7	294 ± 36.3	619 ± 123	822 ± 149	571 ± 48.7	200 ± 38.0	1677 ± 75.5	3874 ± 678	1254 ± 83.8
1376	3-hexen-1-ol	8.91 ± 2.54	9.43 ± 1.44	ND	ND	9.57 ± 0.95	ND	ND	ND	ND	ND	ND	ND
1444	1-heptanol	17.4 ± 2.44	11.9 ± 1.33	10.0 ± 0.73	ND	37.1 ± 4.39	17.9 ± 1.68	24.3 ± 6.99	26.5 ± 0.80	15.7 ± 6.10	16.4 ± 5.28	22.8 ± 2.80	16.2 ± 0.66
1477	2-ethyl-1-hexanol	ND	ND	ND	ND	70.5 ± 2.89	10.4 ± 0.82	15.4 ± 4.60	41.8 ± 1.34	15.3 ± 2.75	18.9 ± 3.70	ND	ND
1532	1,3-butanediol	2895 ± 664	1260 ± 257	1124 ± 181	3457 ± 3398	1382 ± 247	2155 ± 550	2586 ± 766	3387 ± 792	803 ± 354	1653 ± 138	3193 ± 1286	1483 ± 103
1535	2,6-dimethyl-4-heptanol	ND	104 ± 11.4	141 ± 1.3	ND	60.7 ± 25.8	ND	ND	ND	ND	ND	ND	ND
1546	1-octanol	ND	ND	ND	30.4 ± 9.35	12.5 ± 1.71	11.6 ± 1.27	17.6 ± 1.61	16.4 ± 1.55	ND	19.7 ± 8.90	26.3 ± 5.46	13.9 ± 0.83
1565	2,3-butanediol	684 ± 169	199 ± 37.0	205 ± 35.8	1070 ± 561	256 ± 46.0	425 ± 121	506 ± 176	989 ± 302	144 ± 66.6	319 ± 36.1	685 ± 355	251 ± 17.4
1581	propylene glycol	138 ± 31.1	143 ± 29.5	120 ± 19.7	180 ± 109	117 ± 21.5	154 ± 40.2	168 ± 59.2	315 ± 109	58.8 ± 23.0	115 ± 15.4	177 ± 97.4	72.9 ± 4.45
1604	4-methyl-3-hexanol	193 ± 41.8	48.0 ± 8.95	22.4 ± 3.04	27.6 ± 8.63	79.7 ± 37.0	121 ± 47.0	125 ± 47.6	47.0 ± 3.92	76.5 ± 7.81	79.6 ± 5.82	87.8 ± 23.1	65.4 ± 4.64
1649	2-furanmethanol	81.8 ± 34.4	38.9 ± 8.42	22.5 ± 7.85	231 ± 132	199 ± 34.0	152 ± 36.1	324 ± 197	347 ± 30.2	23.7 ± 2.42	1048 ± 307	248 ± 157	144 ± 25.0
1687	2,3-hexanediol	ND	ND	ND	ND	10.3 ± 1.67	7.12 ± 3.19	11.1 ± 4.59	4.89 ± 0.95	ND	ND	ND	ND
1711	methionol	2450 ± 395	1894 ± 191	1930 ± 141	651 ± 196	511 ± 65.0	431 ± 59.0	603 ± 60.6	1263 ± 73.7	631 ± 49.8	1441 ± 218	1818 ± 223	1915 ± 251
1872	phenylmethanol	54.6 ± 16.3	53.4 ± 19.6	14.7 ± 2.00	42.1 ± 22.7	48.5 ± 9.78	56.1 ± 6.11	99.9 ± 7.43	25.2 ± 5.37	70.8 ± 6.65	26.9 ± 2.80	28.3 ± 16.9	38.7 ± 8.93
1919	2-phenylethanol	28,572 ± 4339	24,887 ± 3540	20,061 ± 414	20,658 ± 2895	16,549 ± 1734	20,417 ± 410	22,932 ± 2428	17,507 ± 1758	16,920 ± 1319	20,396 ± 2146	23,663 ± 1692	24,511 ± 973
2310	glycerol	ND	ND	ND	ND	25.7 ± 7.64	75.9 ± 16.1	41.7 ± 17.9	41.1 ± 29.4	ND	ND	ND	ND
2325	2-(4-methoxy phenyl)ethanol	ND	ND	ND	ND	29.1 ± 1.17	11.8 ± 0.96	ND	ND	ND	ND	ND	ND
	**Subtotal**	89,064 ± 7414	87,499 ± 6931	74,368 ± 1642	47,062 ± 9426	76,131 ± 4953	64,769 ± 5420	74,058 ± 3622	69,054 ± 2869	42,411 ± 2563	68,782 ± 3801	79,122 ± 5413	70,847 ± 2334
	***Aldehydes***												
1456	furfural	107 ± 3.15	88.0 ± 9.41	69.4 ± 3.36	69.1 ± 4.76	84.9 ± 8.55	318 ± 20.2	468 ± 104	145 ± 46.8	16.7 ± 0.90	447 ± 31.4	84.8 ± 32.5	189 ± 22.8
2491	5-(hydroxymethyl)-2-furaldehyde	ND	28.0 ± 7.32	39.2 ± 1.78	ND	127 ± 18.4	39.0 ± 7.85	ND	ND	ND	84.2 ± 16.8	ND	ND
	**Subtotal**	107 ± 3.15	116 ± 11.9	109 ± 3.80	69.1 ± 4.76	212 ± 20.3	357 ± 21.7	468 ± 104	145 ± 46.8	16.7 ± 0.90	531 ± 35.6	84.8 ± 32.5	189 ± 22.8
	***Esters***												
946	ethyl propanoate	33.6 ± 3.12	38.7 ± 6.17	17.7 ± 1.22	ND	28.1 ± 10.4	17.6 ± 10.5	ND	ND	ND	32.2 ± 6.42	ND	43.3 ± 1.78
953	ethyl 2-methyl propanoate	ND	40.2 ± 4.19	28.3 ± 2.25	ND	40.2 ± 9.84	4.6 ± 0.85	ND	ND	ND	23.4 ± 8.53	5.32 ± 1.71	18.1 ± 0.90
970	propyl acetate	ND	20.0 ± 2.31	ND	ND	13.4 ± 3.49	26.1 ± 2.95	ND	ND	13.8 ± 8.65	38.4 ± 12.2	ND	31.7 ± 1.98
998	2-methylpropyl acetate	95.7 ± 17.3	59.4 ± 5.64	28.6 ± 1.90	ND	60.7 ± 12.9	55.0 ± 2.19	55.1 ± 4.80	ND	15.6 ± 8.30	110 ± 58.7	71.2 ± 33.0	71.9 ± 5.95
1022	ethyl butanoate	53.6 ± 9.59	57.2 ± 5.79	37.9 ± 2.78	ND	40.6 ± 5.98	38.9 ± 2.13	32.2 ± 4.70	16.2 ± 4.23	6.98 ± 1.31	36.8 ± 8.62	36.9 ± 3.75	61.6 ± 5.02
1039	ethyl 2-methyl butanoate	19.7 ± 3.92	ND	ND	ND	ND	ND	ND	ND	ND	ND	ND	ND
1112	isoamyl acetate	540 ± 115	359 ± 36.1	298 ± 6.71	373 ± 415	365 ± 49.8	486 ± 21.3	467 ± 134	30.4 ± 10.5	100 ± 35.4	807 ± 335	625 ± 142	562 ± 45.3
1125	ethyl pentanoate	ND	ND	ND	ND	ND	5.51 ± 0.19	6.95 ± 0.60	ND	ND	ND	ND	ND
1227	ethyl hexanoate	80.1 ± 10.4	57.0 ± 5.74	50.8 ± 3.94	72.3 ± 62.3	87.0 ± 10.7	51.2 ± 0.96	57.1 ± 18.7	91.3 ± 7.62	11.5 ± 2.17	72.2 ± 21.8	68.0 ± 13.9	82.8 ± 5.19
1258	ethyl pyruvate	49.8 ± 6.56	38.4 ± 3.34	31.1 ± 3.29	ND	19.8 ± 2.20	21.3 ± 2.25	37.7 ± 6.26	12.0 ± 2.08	82.6 ± 6.55	161 ± 21.0	43.8 ± 3.52	78.2 ± 7.34
1309	methyl lactate	ND	132 ± 33.9	46.5 ± 6.77	ND	ND	ND	ND	12.6 ± 0.93	ND	ND	ND	ND
1338	ethyl lactate	5843 ± 864	16,778 ± 1949	11,329 ± 631	45,468 ± 11,102	3536 ± 394	2311 ± 220	2563 ± 182	3585 ± 170	747 ± 62.0	2330 ± 78.8	936 ± 151	1337 ± 76.3
1404	ethyl 2-hydroxy butanoate	9.52 ± 1.78	10.2 ± 0.91	6.77 ± 0.59	11.9 ± 1.60	6.95 ± 1.34	8.49 ± 1.15	10.8 ± 2.01	ND	ND	ND	14.5 ± 2.13	6.98 ± 0.34
1419	ethyl-2-hydroxy-2-methylbutanoate	30.6 ± 5.88	28.7 ± 2.46	27.8 ± 7.79	ND	31.6 ± 4.20	16.2 ± 1.11	19.8 ± 4.20	ND	7.15 ± 0.96	45.5 ± 13.4	ND	19.2 ± 0.72
1424	ethyl octanoate	26.3 ± 3.95	19.3 ± 1.86	18.1 ± 1.69	ND	151 ± 17.2	12.2 ± 0.67	23.5 ± 3.10	ND	34.0 ± 5.66	28.7 ± 9.52	ND	58.2 ± 2.09
1429	ethyl 2-(1-ethoxyethoxy)propanoate	ND	16.0 ± 6.40	5.49 ± 1.71	ND	ND	ND	ND	ND	ND	ND	ND	ND
1451	isobutyl lactate	91.7 ± 10.8	165 ± 5.73	71.2 ± 7.76	128 ± 48.1	ND	ND	ND	146 ± 80.0	13.3 ± 6.67	99.3 ± 5.41	28.8 ± 9.60	ND
1511	ethyl 3-hydroxybutanoate	178 ± 38.6	229 ± 29.4	80.8 ± 6.74	159 ± 46.6	180 ± 20.7	257 ± 32.8	289 ± 54.3	1035 ± 21.5	18.5 ± 2.62	196 ± 23.8	205 ± 31.3	243 ± 12.2
1562	isoamyl lactate	138. ± 21.7	261. ± 25.7	144. ± 20.9	446. ± 203	55.6 ± 9.06	30.6 ± 2.61	37.4 ± 10.46	68.8 ± 5.53	11.6 ± 2.60	120 ± 94.1	49.0 ± 18.10	31.6 ± 0.57
1626	ethyl methyl succinate	35.7 ± 5.31	ND	ND	21.8 ± 9.27	ND	9.14 ± 2.86	14.7 ± 1.98	ND	ND	ND	47.8 ± 8.72	38.8 ± 3.19
1665	diethyl succinate	5462 ± 1207	456 ± 50.1	491 ± 42.5	8015 ± 1590	344 ± 53.8	255 ± 10.1	371 ± 22.4	1183 ± 85.2	199 ± 42.2	411 ± 127	317 ± 5.98	483 ± 23.9
1669	ethyl 3-hydroxyhexanoate	ND	109 ± 16.8	49.2 ± 9.78	ND	73.5 ± 11.9	26.3 ± 11.0	46.1 ± 18.6	20.7 ± 7.02	24.3 ± 3.62	53.5 ± 3.56	31.3 ± 7.69	23.7 ± 6.00
1782	ethyl phenylacetate	28.0 ± 14.0	59.6 ± 36.9	39.4 ± 25.3	61.2 ± 16.3	27.8 ± 18.3	17.2 ± 5.70	13.7 ± 7.86	ND	ND	ND	ND	ND
1799	ethyl 4-hydroxybutanoate	4225 ± 635	3229 ± 860	2905 ± 119	1454 ± 249	3713 ± 426	4079 ± 655	4698 ± 522	3958 ± 376	2091 ± 814	3455 ± 307	1916 ± 214	3002 ± 463
1815	2-phenylethyl acetate	378 ± 67.6	ND	180 ± 35.7	125 ± 71.1	118 ± 36.5	600 ± 25.9	759 ± 41.7	26.9 ± 6.11	188 ± 67.5	538 ± 139.3	557 ± 109	257 ± 29.7
1899	ethyl 3-methylbutyl succinate	47.2 ± 12.3	1287 ± 189	1171 ± 53.1	101 ± 47.9	128 ± 14.7	10.9 ± 2.66	19.8 ± 3.16	13.7 ± 3.04	26.3 ± 3.24	39.6 ± 38.3	47.2 ± 12.3	1287 ± 189
1997	methyl 2-furoate	64.3 ± 13.9	51.8 ± 6.98	41.9 ± 2.67	ND	70.7 ± 10.7	13.6 ± 1.05	16.6 ± 2.29	19.6 ± 9.42	ND	186 ± 32.0	64.3 ± 13.9	51.8 ± 6.98
2031	diethyl malate	120 ± 33.3	133 ± 41.2	161 ± 12.2	ND	ND	ND	ND	ND	100 ± 15.4	36.8 ± 7.37	120 ± 33.3	133 ± 41.2
2098	diethyl 2-hydroxypentanedioate	43.3 ± 12.0	117 ± 17.0	85.5 ± 12.9	ND	43.5 ± 6.64	22.6 ± 1.90	22.7 ± 6.90	ND	11.8 ± 2.49	32.0 ± 19.7	43.3 ± 12.0	117 ± 17.0
2278	ethyl 2-hydroxy-3-phenyl propanoate	313 ± 21.6	112 ± 13.8	157 ± 4.37	416 ± 58.1	119 ± 10.4	144 ± 12.3	152 ± 54.1	43.1 ± 16.3	298 ± 71.6	139 ± 28.0	82.3 ± 20.2	273 ± 43.4
2367	ethyl hydrogen succinate	5490 ± 772	2504 ± 483	3442 ± 245	6056 ± 622	4328 ± 284	2009 ± 171	1956 ± 826	1268 ± 544	712 ± 260	720 ± 115	250 ± 64.2	887 ± 147
2454	ethyl citrate	ND	ND	ND	ND	ND	ND	ND	ND	ND	34.1 ± 10.0	ND	ND
	**Subtotal**	23,396 ± 1796	26,367 ± 2195	20,944 ± 693	62,908 ± 11,246	13,580 ± 652	10,527 ± 714	11,669 ± 1007	11,530 ± 694	4713 ± 865	9744 ± 528	5333 ± 329	7707 ± 498
	***Furans***												
1453	2-(diethoxymethyl)furan	73.4 ± 22.7	ND ^3^	41.3 ± 7.99	ND	135 ± 21.3	235 ± 107	130 ± 77.7	ND	ND	ND	203 ± 136	370 ± 13.9
1498	2-acetylfuran	38.1 ± 9.28	14.0 ± 0.69	12.5 ± 1.05	ND	16.0 ± 2.10	7.47 ± 0.97	10.0 ± 2.11	ND	30.0 ± 8.24	35.5 ± 17.8	36.1 ± 37.0	17.7 ± 1.01
	**Subtotal**	112 ± 24.6	14.0 ± 0.69	53.8 ± 8.06	ND	151 ± 21.4	242 ± 107	140 ± 77.7	ND	30.0 ± 8.24	35.5 ± 17.8	239 ± 141	388 ± 14.0
	***Ketones***												
971	2,3-butanedione	70.8 ± 11.8	ND	14.2 ± 3.68	27.3 ± 19.5	ND	26.7 ± 3.69	ND	33.6 ± 21.0	41.3 ± 25.3	24.7 ± 12.6	27.8 ± 10.8	27.6 ± 3.39
1116	3-penten-2-one	19.4 ± 2.77	36.6 ± 6.23	29.6 ± 0.84	ND	8.30 ± 2.38	29.7 ± 5.01	16.8 ± 3.28	ND	ND	16.8 ± 1.16	9.82 ± 0.70	21.2 ± 3.56
1280	3-hydroxy-2-butanone (acetoin)	868 ± 103	233 ± 24.2	58.4 ± 6.73	135 ± 75.3	114 ± 13.4	33.2 ± 5.00	76.7 ± 13.3	153 ± 12.8	728 ± 101	129 ± 8.78	246 ± 38.9	99.2 ± 13.1
1294	1-hydroxy-2-propanone (acetol)	93.2 ± 14.8	48.1 ± 18.9	35.0 ± 13.9	ND	64.9 ± 8.79	9.13 ± 3.73	8.36 ± 1.69	18.8 ± 7.88	190 ± 29.6	66.4 ± 33.0	70.3 ± 23.8	18.4 ± 9.63
1343	3,3,6-trimethyl-1,5-heptadien-4-one	ND	ND	ND	ND	ND	ND	ND	ND	ND	ND	141 ± 31.9	ND
1721	3-methyl-2(5 H)-furanone	ND	ND	ND	ND	56.7 ± 7.34	ND	ND	ND	9.57 ± 0.57	ND	7.82 ± 0.67	9.61 ± 1.38
1736	piperitone	ND	ND	ND	ND	25.3 ± 7.28	ND	ND	ND	ND	ND	ND	ND
1825	tetrahydro-4-methyl-2H-pyran-2-one	ND	ND	ND	16.4 ± 1.45	ND	27.7 ± 2.11	ND	41.9 ± 7.64	ND	24.5 ± 6.50	57.5 ± 2.59	62.0 ± 7.78
1969	maltol	ND	ND	ND	41.3 ± 24.0	ND	ND	ND	ND	ND	ND	ND	ND
2042	5-acetyldihydro-2(3H)-furanone	ND	ND	ND	ND	16.0 ± 2.28	ND	ND	ND	ND	ND	ND	ND
2473	5-hydroxymethyl dihydrofuran-2-one	41.0 ± 13.0	19.8 ± 8.30	45.2 ± 9.52	ND	ND	ND	29.6 ± 6.46	ND	ND	ND	ND	ND
	**Subtotal**	1093 ± 105	338 ± 32.4	182 ± 18.6	220 ± 81.4	285 ± 19.4	126 ± 9.07	131 ± 15.2	247 ± 26.9	969 ± 109	262 ± 37.0	560 ± 56.7	238 ± 18.7
	***Lactones***												
1637	γ-butyrolactone	2386 ± 530	2782 ± 232	2813 ± 208	544 ± 156	2874 ± 415	ND	2161 ± 121	596 ± 58.7	580 ± 200	3130 ± 270	300 ± 34.3	993 ± 21.4
1707	γ-hexalactone	ND	ND	ND	12.4 ± 3.39	11.5 ± 1.31	13.5 ± 1.22	12.4 ± 0.99	13.0 ± 1.08	ND	ND	17.5 ± 1.99	14.4 ± 1.95
1725	γ-ethoxybutyrolactone	22.3 ± 6.66	ND	24.0 ± 2.98	11.0 ± 1.45	76.4 ± 9.84	ND	ND	21.7 ± 4.22	10.5 ± 3.74	ND	36.7 ± 6.16	ND
2026	pantolactone	55.3 ± 9.02	74.1 ± 12.3	ND	51.5 ± 7.13	248 ± 37.5	113 ± 10.8	193 ± 71.6	57.3 ± 18.1	ND	ND	85.0 ± 34.1	203 ± 15.0
2032	γ-nonalactone	ND	ND	ND	83.9 ± 17.0	112 ± 15.3	37.1 ± 0.99	52.6 ± 14.5	50.4 ± 16.3	ND	5151 ± 1094	14.7 ± 3.71	18.5 ± 1.56
2230	4-ethoxycarbonyl-γ-butyrolactone	128 ± 35.3	90.0 ± 8.35	101 ± 7.77	34.6 ± 3.65	112 ± 11.1	60.6 ± 3.21	66.8 ± 20.4	22.4 ± 8.26	26.2 ± 7.40	57.7 ± 11.5	ND	43.4 ± 6.49
2377	4-(1-hydroxyethyl)-γ-butyrolactone	65.9 ± 14.9	33.8 ± 12.9	39.1 ± 4.03	159 ± 23.2	127 ± 16.6	67.7 ± 15.3	69.7 ± 31.7	65.9 ± 28.1	19.6 ± 8.22	ND	ND	21.0 ± 3.99
	**Subtotal**	2657 ± 532	2980 ± 233	2977 ± 209	896 ± 159	3562 ± 417	291 ± 19.1	2555 ± 146	827 ± 70.2	637 ± 201	8339 ± 1127	454 ± 48.9	1294 ± 27.3
	***Phenols***												
1857	guaiacol	ND	14.6 ± 3.81	ND	ND	ND	ND	ND	11.9 ± 6.64	23.5 ± 13.4	17.2 ± 6.01	ND	ND
1983	phenol	ND	ND	11.2 ± 1.44	58.9 ± 8.54	21.6 ± 7.85	10.5 ± 0.11	13.3 ± 0.61	15.4 ± 2.79	8.51 ± 0.95	11.2 ± 2.90	17.0 ± 3.06	27.4 ± 4.41
2016	4-ethylguaiacol	ND	ND	ND	559 ± 69.4	ND	ND	ND	ND	ND	ND	ND	ND
2063	4-methylphenol	ND	ND	ND	38.6 ± 7.06	ND	ND	ND	ND	ND	12.9 ± 5.0	ND	ND
2165	4-ethylphenol	ND	ND	ND	88.6 ± 9.39	ND	ND	ND	ND	ND	ND	ND	ND
2192	2-methoxy-4-vinylphenol	120 ± 18.8	326 ± 94.8	58.1 ± 16.9	50.8 ± 19.5	21.5 ± 7.57	29.4 ± 4.37	58.2 ± 19.4	369 ± 82.7	ND	68.6 ± 18.4	52.7 ± 23.1	202 ± 18.4
2203	2-methyl-5-(1-methylethyl) phenol	ND	ND	ND	ND	ND	11.7 ± 1.70	ND	21.9 ± 9.40	ND	28.1 ± 11.5	ND	35.1 ± 6.53
2382	4-vinylphenol	111 ± 42.0	73.1 ± 26.6	85.4 ± 8.02	ND	ND	ND	ND	ND	ND	ND	ND	36.8 ± 2.12
	**Subtotal**	231 ± 46.0	414 ± 98.5	155 ± 18.8	796 ± 73.5	43.1 ± 10.9	51.6 ± 4.69	71.5 ± 19.4	418 ± 83.5	32.0 ± 13.5	138 ± 23.3	69.7 ± 23.3	301 ± 20.2
	***Terpenoids***												
1223	eucalyptol	26.1 ± 6.87	ND	ND	ND	120 ± 5.21	ND	ND	25.2 ± 7.50	ND	24.6 ± 11.7	38.6 ± 2.63	ND
1600	4-terpineol	ND	ND	7.46 ± 0.80	ND	52.5 ± 11.0	15.5 ± 8.13	17.3 ± 9.28	ND	ND	ND	24.2 ± 10.7	53.6 ± 10.1
1692	α-terpineol	9.08 ± 1.10	ND	8.86 ± 0.82	19.2 ± 4.04	12.9 ± 1.25	ND	ND	ND	ND	52.7 ± 10.3	ND	41.9 ± 5.41
1701	borneol	15.9 ± 2.01	ND	ND	ND	133 ± 15.1	ND	ND	ND	ND	25.1 ± 4.34	ND	ND
1842	*p*-cymen-8-ol	ND	ND	ND	ND	14.6 ± 1.93	ND	9.38 ± 2.64	ND	ND	ND	18.7 ± 3.41	27.6 ± 0.72
2073	*p*-cymen-7-ol	ND	ND	ND	ND	12.7 ± 2.0	ND	ND	ND	ND	ND	ND	ND
	**Subtotal**	51.1 ± 7.24	ND	16.3 ± 1.15	19.2 ± 4.04	346 ± 19.6	15.5 ± 8.13	26.7 ± 9.65	25.2 ± 7.50	ND	102 ± 16.2	81.4 ± 11.5	123 ± 11.5
	***Miscellaneous***												
969	1-(1-ethoxyethoxy) propane	18.8 ± 10.7	47.5 ± 18.6	18.3 ± 6.18	ND	24.7 ± 8.26	11.9 ± 1.93	15.0 ± 0.90	ND	8.12 ± 1.20	34.2 ± 19.2	56.4 ± 13.5	25.6 ± 4.82
974	2,4,5-trimethyl-1,3-dioxolane	10.7 ± 1.90	10.9 ± 2.55	ND	ND	5.62 ± 1.22	ND	9.66 ± 4.54	ND	14.1 ± 5.28	12.5 ± 8.12	26.3 ± 19.4	ND
987	1-(1-ethoxyethoxy) butane	111 ± 66.2	387 ± 165	117 ± 42.4	ND	199 ± 66.3	49.9 ± 10.7	44.3 ± 14.7	15.2 ± 9.02	192 ± 118	109 ± 26.0	80.0 ± 24.1	106 ± 21.5
1053	2-methyl-1,3-dioxane	15.7 ± 2.83	25.7 ± 3.20	15.7 ± 5.18	ND	ND	ND	ND	ND	9.46 ± 4.96	16.5 ± 4.62	ND	7.91 ± 2.47
1068	1,1-diethoxy-2-methylbutane	ND	37.3 ± 22.6	12.9 ± 3.26	20.8 ± 17.8	17.0 ± 8.31	83.1 ± 6.08	21.2 ± 6.64	21.8 ± 12.4	13.1 ± 6.88	ND	12.8 ± 7.53	17.7 ± 1.91
1069	1,1-diethoxy-3-methylbutane	21.5 ± 7.31	39.8 ± 10.3	19.6 ± 3.18	49.4 ± 28.4	28.7 ± 4.86	20.2 ± 2.28	51.4 ± 38.1	142 ± 88.1	53.6 ± 20.5	21.8 ± 14.0	36.9 ± 16.6	54.0 ± 7.11
1098	1-(1-ethoxyethoxy) pentane	262 ± 171	817 ± 366	338 ± 130	ND	540 ± 183	164 ± 43.7	169 ± 91.2	41.8 ± 25.1	598 ± 367	279 ± 64.7	248 ± 103	268 ± 51.6
1225	1,1-diethoxyhexane	18.9 ± 3.33	12.1 ± 2.15	13.7 ± 1.83	ND	31.6 ± 3.99	ND	50.5 ± 23.6	ND	ND	ND	ND	10.4 ± 0.39
1494	5-hydroxy-2-methyl-1,3-dioxane	66.3 ± 14.1	195 ± 31.1	115 ± 16.4	ND	126 ± 18.5	27.9 ± 6.90	48.3 ± 17.9	8.43 ± 0.10	21.9 ± 2.76	86.8 ± 6.32	24.3 ± 4.32	25.1 ± 2.78
1629	ethyl acetamide	10.7 ± 1.91	11.5 ± 1.70	11.7 ± 6.86	ND	33.1 ± 13.7	7.30 ± 0.53	9.27 ± 0.39	10.9 ± 3.59	ND	21.6 ± 11.2	ND	ND
1862	N-(3-methylbutyl) acetamide	22.9 ± 13.5	24.1 ± 9.49	ND	ND	16.3 ± 8.18	15.3 ± 5.51	47.0 ± 42.2	38.9 ± 5.69	12.8 ± 1.57	ND	54.4 ± 30.7	57.8 ± 8.15
1970	2-acetylpyrrole	ND	ND	ND	ND	ND	ND	10.0 ± 2.17	11.4 ± 2.00	ND	11.3 ± 2.51	ND	ND
	**Subtotal**	558 ± 184	1607 ± 404	661 ± 138	70.2 ± 33.5	1022 ± 197	380 ± 46.3	475 ± 113	290 ± 93.2	923 ± 386	593 ± 75.4	540 ± 114	573 ± 57.3
	**Total**	120,390 ± 7658	123,505 ± 7301	102,204 ± 1808	130,811 ± 14,792	99,108 ± 5023	79,030 ± 5479	91,968 ± 3794	88,966 ± 2972	51,925 ± 2767	91,550 ± 4006	90,364 ± 5443	83,795 ± 2390

^1^ Mean value of 3 replications ± SD. ^2^ RI (Retention indices) were determined on DB-wax using C6-C26 as external reference. ND stands for not detected.
